# Children of a syndemic: co‐occurring and mutually reinforcing adverse child health exposures in a prospective cohort of HIV‐affected mother‐infant dyads in Cape Town, South Africa

**DOI:** 10.1002/jia2.26152

**Published:** 2023-11-01

**Authors:** Stanzi M. le Roux, Elaine J. Abrams, Allison Zerbe, Tamsin K. Phillips, Landon Myer

**Affiliations:** ^1^ Division of Epidemiology & Biostatistics School of Public Health, University of Cape Town Cape Town South Africa; ^2^ Mailman School of Public Health ICAP at Columbia University, Columbia University New York New York USA; ^3^ Department of Pediatrics, Vagelos College of Physicians & Surgeons Columbia University New York New York USA

**Keywords:** HIV, perinatology, vertical transmission, syndemic, social determinants of health, Africa

## Abstract

**Introduction:**

Several HIV‐related syndemics have been described among adults. We investigated syndemic vulnerability to hazardous drinking (HD), intimate partner violence (IPV) and household food insecurity (HFIS) in breastfed children born without HIV in urban South Africa. We compared those who were perinatally HIV exposed (CHEU) to those who were not (CHU), under conditions of universal maternal antiretroviral therapy (ART) and breastfeeding.

**Methods:**

A prospective cohort of pregnant women living with HIV (WLHIV), and without HIV, were enrolled and followed with their infants for 12 months postpartum (2013–2017). All WLHIV initiated antenatal efavirenz‐based ART. Measurements of growth (∼3 monthly), infectious cause hospitalisation, ambulatory childhood illness (2‐week recall) and neurodevelopment (BSID‐III, measured at ∼12 months’ age) were compared across bio‐social strata using generalised linear regression models, with interaction terms; maternal data included interview‐based measures for HD (AUDIT‐C), IPV (WHO VAW) and HFIS.

**Results:**

Among 872 breastfeeding mother‐infant pairs (*n* = 461 CHEU, *n* = 411 CHU), WLHIV (vs. HIV negative) reported more unemployment (279/461, 60% vs. 217/411, 53%; *p* = 0.02), incomplete secondary education (347/461, 75% vs. 227/411, 55%; *p* < 0.0001), HD (25%, 117/459 vs. 7%, 30/411; *p* < 0.0001) and IPV (22%, 101/457 vs. 8%, 32/411; *p* < 0.0001) at enrolment; and HFIS at 12 months (45%, 172/386 vs. 30%, 105/352; *p* > 0.0001). There were positive interactions between maternal HIV and other characteristics. Compared to food secure CHU, the mean difference (95% CI) in weight‐for‐age Z‐score (WAZ) was 0.06 (−0.14; 0.25) for food insecure CHU; −0.26 (−0.42; −0.10) for food secure CHEU; and −0.43 (−0.61; −0.25), for food insecure CHEU. Results were similar for underweight (WAZ < −2), infectious‐cause hospitalisation, cognitive and motor delay. HIV‐IPV interactions were evident for ambulatory diarrhoea and motor delay. There were HIV‐HD interactions for odds of underweight, stunting, cognitive and motor delay. Compared to HD‐unexposed CHU, the odds ratios (95% CI) of underweight were 2.31 (1.11; 4.82) for HD‐exposed CHU; 3.57 (0.84; 15.13) for HD‐unexposed CHEU and 6.01 (2.22; 16.22) for HD‐exposed CHEU.

**Conclusions:**

These data suggest that maternal HIV‐related syndemics may partly drive excess CHEU health risks, highlighting an urgent need for holistic maternal and family care and support alongside ART to optimise the health of CHEU.

## 1 INTRODUCTION

In 2021, the global population of children who are perinatally HIV exposed but HIV negative (CHEU) was almost 16 million, with another one million CHEU born annually [1, 2]. The largest number of CHEU live in sub‐Saharan Africa, predominantly in South Africa where an estimated 20% of the under‐15 population are CHEU [1, 3]. Prior to the widespread availability of universal maternal antiretroviral therapy (ART), predominantly formula‐fed CHEU were reported to have excess risks of mortality, infectious morbidity, growth restriction and developmental delays compared to HIV‐negative children who are perinatally HIV unexposed (CHU) [4, 5]. Emerging data suggest that while the magnitude of these historic risk differences is substantially diminished under conditions of universal maternal ART with optimal breastfeeding, some risk differential remains [6, 7, 8, 9, 10]. In regions with large numbers of CHEU, even small increases in risk are likely to adversely impact child health at a population level [9]. Understanding the drivers of these residual differences is necessary for the creation and implementation of effective public health interventions, in turn promoting child health in high HIV burden settings.

The syndemic model of health seeks to assess the co‐occurrence of and interactions between social determinants of health, biological factors and disease outcomes within the context of inequality and deprivation [11]. Reflecting the high incidence of HIV among people who are marginalised and poor, several HIV‐related syndemics have been described among women living with HIV (WLHIV), including inter‐relationships between hazardous use of alcohol, household food insecurity (HFIS) and intimate partner violence (IPV), which in turn exacerbate HIV disease severity, adversely affect adherence to ART and increase the risk of mental health challenges [12, 13, 14]. Each of these maternal factors is also known to increase the risks of adverse health outcomes among children globally, including CHEU [15, 16, 17]. In a prospective cohort study comparing breastfeeding CHEU to CHU over the first year of life in the context of universal, maternal efavirenz‐based ART, we previously reported small but clinically meaningful increased risks of growth restriction, early infectious cause hospitalisation, ambulatory childhood illness, cognitive delay and motor delay [6, 7, 8]. Previously, we noted the independent effects of individual psychosocial, behavioural and economic factors on child health outcomes. This secondary analysis investigates the potential role of maternal HIV‐related syndemic relationships in the previously reported adverse health outcomes among CHEU (Figure S1).

## 2 METHODS

### 2.1 Study design and enrolment

We conducted a secondary analysis of data collected in two linked, prospective cohort studies, conducted in parallel at the Gugulethu Midwife and Obstetric Unit, a primary care centre in an impoverished community of peri‐urban Cape Town, South Africa (enrolment, June 2013–April 2016; follow‐up through March 2017). At the first antenatal visit, the MCH‐ART (**M**aternal and **C**hild **H**ealth **A**nti‐**R**etroviral **T**herapy) study enrolled WLHIV who were initiating ART during pregnancy; the HU2 (**H**IV‐**u**nexposed **u**ninfected) study enrolled pregnant women who tested HIV negative. Details regarding the study methodology have been published elsewhere [6, 7, 8, 18]. Briefly, women were followed up through delivery, and with their HIV‐negative, breastfeeding infants, until at least 12 months postpartum (visits at 6 weeks; 3 monthly from 3 to 12 months). Apart from HIV‐specific measures, study follow‐up, procedures and measurements were identical for both studies, utilising the same study site, materials and staff.

### 2.2 Study measurements

#### 2.2.1 Maternal measures

At enrolment and during follow‐up, demographic, psychosocial and behavioural data were collected by trained study counsellors administering standardised, locally validated, questionnaires in private rooms. All questionnaires were translated into both isiXhosa and Afrikaans, and administered in the participant's language of choice. We used the Alcohol Use Disorders Identification Test (AUDIT) [19] to measure maternal alcohol use (“hazardous drinking,” HD defined as AUDIT consumption score ≥3); the Edinburgh Postnatal Depression Scale [20] to estimate probable maternal depression (EPDS score ≥13); and the World Health Organisation Violence against Women (VAW) questionnaire for IPV (any psychological, physical or sexual violence) [21]. At the 12‐month visit, we also assessed HFIS (defined as “has, or is at risk of food insecurity” vs. no food insecurity) using a questionnaire (reference period, “ever”) previously used in South Africa, adapted from the Household Food Insecurity Access Scale, Food and Nutrition Technical Assistance Project and the Community Childhood Hunger Identification Project Index [22]. To minimise participant time commitments for interim study visits, the psychosocial/behavioural tools (AUDIT, EPDS and VAW) were only administered at pre‐specified visits. This analysis focuses primarily on potential syndemic factors measured at the first antenatal visit, and at 12 months postpartum, the only time points when all three of these tools were administered at the same study visit (other time points of measurement are shown in tables).

#### 2.2.2 Child health measures

Measurement and quality assurance information have previously been published in detail for each of the key child health outcomes [6, 7, 8]. In summary, trained counsellors conducted supervised anthropometric measurements using calibrated tools at each study visit [6]. We used anthropometric software (Intergrowth‐21st Project and WHO Multicentre Growth Reference Study as indicated) to generate Z‐scores (adjusted for age, sex and gestation) for weight‐for‐age (WAZ), length‐for‐age (LAZ) and head circumference‐for‐age (HCAZ). We measured infectious morbidity through (1) hospitalisation data abstracted from Provincial Health Data Centre databases [23] with diagnoses based on the International Classification of Diseases, 10th edition; and (2) point prevalence (2‐week recall) of ambulatory childhood illness (presumed lower respiratory tract illness, RTI; diarrhoeal illness) at each study visit, using questions from the Demographic and Health Survey (DHS) [7, 24]. A trained clinician assessed neurodevelopment using the Bayley Scales of Infant Development (3rd edition, BSID‐III) on a subset of study participants at approximately 12 months of age [8, 25, 26].

### 2.3 Statistical methodology

Sample size calculations for the primary analyses were based on direct comparisons of CHEU to CHU with >80% power to detect *a priori*‐defined clinically meaningful differences (definitions and achieved power shown in Table S1) [6, 7, 8].

A syndemic is defined as “population‐level clustering of social and health problems,” with three main characteristics: (a) the presence of adverse contextual and social factors which drive disease concentration; (b) the clustering of diseases within a specific population; and (c) adverse disease interaction, where concurrent (bidirectional or co‐occurring) factors (biological, social or behavioural) increase morbidity or mortality [11]. Accordingly, we described the social circumstances of our study population, in which context the following inter‐related hypotheses were tested, derived from syndemic theory (Figure S1):

- HD, IPV and HFIS are more common among women who are HIV positive than women who are HIV negative (clustering of potential syndemic factors)
- Maternal HIV, HD, IPV and HFIS have independent adverse effects on the health of CHEU and CHU (direct effects on child health)
- There is positive interaction (synergistic risk exacerbation of adverse child health outcomes) between maternal HIV and at least one of the examined co‐factors (HD, IPV or HFIS), which result in differences that may have an impact on either an individual or population level, for at least one of the following outcomes (“potentially meaningful” differences defined below):
  - Child growth (>0.2 absolute difference in Z‐score, or relative increase of >1.2 for Z‐score < 2)
  - Infectious morbidity (odds ratio or risk ratio of >1.5 for any of the defined measures)
  - Neurodevelopmental delay (absolute difference of >5 composite score, or relative increase of >1.2 in the odds ratio for cognitive or motor delay, defined as composite score < 85)

We used standard data exploration techniques and generalised linear regression models with outcome‐specific link functions, correcting for repeat measures with random effects models or generalised estimating equations. Approaches followed those utilised in the original publications where possible [6, 7, 8]. In all original analyses, choices of third variables were based on *a priori*‐specified directed acyclic graphs; where more than one variable measuring the same construct was available, we were guided by Akaike's Information Criterion. The HFIS questionnaire we utilised categorised households as being “not at risk,” “at risk for food insecurity” or “food insecure.” In exploratory analyses, there were little differences between the “at risk” and “food insecure” groups; for best model fit, we created a binary variable combining these two groups. To accommodate multiple imputation for infectious morbidity analyses, we used logistic regression analysis for dichotomous hospitalisation and ambulatory infectious illness variables, a simplified approach compared to the analyses used in the original publication. We used both interaction (product) terms and indicator variables in regression analyses to test hypothesis (iii), and calculated Rothman's interaction contrast (IC) and relative excess risk due to interaction (RERI_RR_) to clearly demonstrate the direction and scale of interactions [27]. All analyses were completed using Stata 16 (StataCorp); we also used GraphPad Prism version 9.0 (GraphPad Software, www.graphpad.com) and DeepVenn software for additional data visualizstion [28].

#### 2.3.1 Missing data

Less than 1% of mother‐child pairs had at least one potential syndemic factor missing at the antenatal enrolment visit; 16% (137/872) had at least one factor missing at the 12 months’ postpartum visit, including food security data. Differences by the availability of HFIS data are shown in Table S2, stratified by maternal HIV status. To maximise efficiency, we used multiple imputation (chained equations) [29] in the Stata 16 multiple imputation package (StataCorp) to impute 20 datasets for regression modelling (details shown in Table S3), combining analytic estimates using Rubin's rules [30].

### 2.4 Ethics

The University of Cape Town Faculty of Health Sciences’ Research Ethics Committee approved the MCH‐ART and HU2 studies. Both studies conform to the Declaration of Helsinki; all women provided written, informed consent.

## 3 RESULTS

Overall, 872 mother‐child pairs contributed to this analysis (CHEU, *n* = 461; CHU, *n* = 411); 717 (82%) completed 12 months of postnatal follow‐up (Figure S2). There were minimal differences between those who contributed and did not contribute data at 12 months (Table S2).

Overall, most women were living in informal housing, without running water and/or flushable toilets inside the home at study enrolment (Table 1). Compared to HIV‐negative women, WLHIV were more likely to be unemployed (279/461, 60% vs. 217/411, 53%; *p* = 0.02) and have incomplete secondary education (347/461, 75% vs. 227/411, 55%; *p* < 0.0001, Table 1). Co‐distributions of maternal factors are shown in Table S3.

**Table 1:** Maternal and infant characteristics, by maternal HIV status

	Total (*N* = 872)	Women who are HIV positive, and CHEU (*n* = 461)	Women who are HIV negative, and CHU (*n* = 411)	Missing, pre‐imputation	Missing, after imputation^a^
**MATERNAL CHARACTERISTICS**					
**Demographics and household characteristics**					
Age in years, mean (SD)	28 (6)	28 (5)	27 (6)	0	n/a
Married/cohabiting	373 (43%)	189 (41%)	184 (45%)	0	0
Incomplete secondary education	574 (66%)	347 (75%)	227 (55%)	0	0
Unemployed	496 (57%)	279 (60%)	217 (53%)	0	0
Informal housing	438 (50%)	242 (52%)	196 (48%)	0	0
No flushable toilet inside home	580 (67%)	335 (73%)	245 (60%)	0	0
No running water inside home	468 (54%)	272 (59%)	196 (48%)	0	0
Lives in a formal, brick home, with flushable toilet and running water inside the home					
*No, lacks at least one of the above*	592 (68%)	336 (73%)	256 (62%)		
*Yes, has all three*	280 (32%)	125 (27%)	155 (38%)		
Household crowding (≥10 people)	40 (5%)	28 (6%)	12 (3%)	0	0
**Maternal HIV‐related measures at enrolment and delivery**					
CD4 cell count at ART initiation (cells/mm^3^)	–	354 (249–527)	–	12	n/a
Log_10_ HIV viral load at ART initiation (copies/ml)	–	4.0 (3.3–4.5)	–	0	n/a
Log_10_ HIV viral load at delivery (copies/ml)		1.6 (1.6–1.6)		0	n/a
HIV viral load <50 copies/ml at delivery	–	352 (76%)	–	0	n/a
**INFANT CHARACTERISTICS**					
Gestational age at ART initiation (weeks)	–	22 (17–27)	–	0	n/a
Gestational age at delivery (weeks)	39 (38–40)	39 (38–40)	39 (38–40)	0	0
*Preterm (<37)*	94 (11%)	56 (12%)	38 (9%)	0	n/a
Weight‐for‐age Z‐score at birth	−0.13 (−0.84; 0.51)	−0.21 (−0.93; 0.37)	−0.05 (−0.72; 0.64)	0	0
Small‐for‐gestational‐age (birthweight <10th centile)^b^	90 (10%)	51 (11%)	39 (10%)	0	n/a
Male sex	428 (49%)	232 (50%)	196 (48%)	0	n/a
Early introduction of breastfeeding (within first hour of life)^c^	788/867 (91%)	400/459 (87%)	388/408 (95%)	5	0
Duration of EBF (months)^c^	1.4 (0.1–3.0)	1.4 (0.2–3.5)	1.2 (0.1–3.0)	0	0
Duration of any breastfeeding (months)^c^	6.1 (1.5–12.0)	3.9 (1.4–12.0)	9.0 (3.0–12.0)	0	0

*Note*: Results are *n* (column %) with *p*‐value from chi^2^ test; mean (SD) with *p*‐value from *t*‐test for normally distributed variables; or median (interquartile range, IQR) with *p*‐value from Kruskal–Wallis.

Abbreviations: ART, antiretroviral therapy; CHEU, children who were perinatally HIV exposed but are HIV negative; CHU, children who were perinatally HIV unexposed and are HIV negative; EBF, exclusive breastfeeding; ml, millilitre; mm^3^, cubic millimetre; SD, standard deviation.

^a^n/a, not applicable (variables not included in imputation model); post imputation missing values not used if maternal or child death occurred.

^b^Birth weight percentile based on Intergrowth‐21st reference standards.

^c^Maternal report (24‐hour recall); exclusive breastfeeding defined as only breast milk and prescribed medicine.

### 3.1 Clustering of potential maternal syndemic factors at enrolment and over time

**Figure 1:**
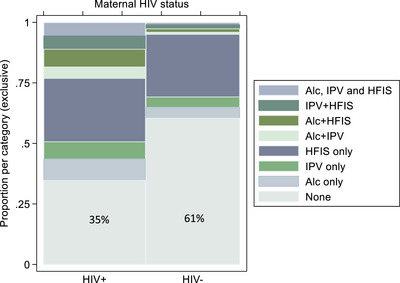
Spine plot showing distributions of maternal hazardous drinking (“Alc”), intimate partner violence (IPV), and household food insecurity (HFIS), by maternal HIV status.

At antenatal enrolment, WLHIV (vs. HIV‐negative women) reported more HD (25% [117/459] vs. 7% [30/411], *p* < 0.0001) and IPV (22% [101/457] vs. 8% [32/411], *p* < 0.0001), Table 2. One in three WLHIV with HD also reported concurrent IPV (38%, 44/116), compared to one in five of WLHIV without HD (17%, 57/341); absolute risk difference (RD) 21% (95% CI 11%–31%). This difference was smaller among HIV‐negative women (prevalence of IPV among women reporting any vs. no HD, 20% vs. 7%; RD 13%, 95% CI −1% to 28%). For both HD and IPV independently, risks at enrolment predicted risks at 12 months postpartum: the prevalence of HD at 12 months (12% overall, Table 2) was five times higher among women who had also reported any (vs. no) HD at enrolment (37% vs. 7%; relative risk, RR 5.27; 95% CI 3.66–7.59); similar results were seen for IPV at 12 months (comparing those with vs. without IPV at enrolment: RR 4.0, 95% CI 2.61–6.27), and when stratified by HIV status (data not shown). At enrolment and over time, the prevalence of probable or possible maternal depression (as measured by the EPDS at threshold values of 13, and of 10) did not vary substantially by HIV status (Table 2). At 12 months, 45% (172/386) of WLHIV experienced or were at risk of HFIS, compared to 30% (105/352) of HIV‐negative women (*p* < 0.0001, Table 2). WLHIV were notably more likely to report frequent household hunger (due to lack of resources) than HIV‐negative women (based on responses to the three questions directly addressing hunger: 72/386, 19% vs. 6/352, 2%), suggesting severe food insecurity [31]. At 12 months, concurrent HD or IPV predicted increased risks of HFIS: the relative risk for HFIS was 1.56 (95% CI 1.26–1.94) when HD was also reported; and 1.78 (95% CI 1.43–2.20) for HFIS when IPV was also reported. Two‐thirds of WLHIV had at least one of the potential syndemic factors (HD, IPV or HFIS), compared to only a third of HIV‐negative women (Table 2 and Figure 1). Proportional Venn diagrams for the overlap of these factors are shown in Figures S3a–c.

**Table 2:** Maternal psychosocial and behavioural factors at antenatal enrolment and over time, by maternal HIV status^a^

	Total (*N* = 872)	Women who are HIV positive (*n* = 461)	Women who are HIV negative (*n* = 411)	*p‐*value	Missing, pre‐imputation	Missing, after imputation^b^
**Alcohol Use Disorder Identification Tool (AUDIT) measurements**						
Total score						
*At first antenatal visit, study enrolment* ^c^	0 (0–1)	0 (0–4)	0 (0–0)	0.0001	2	n/a
*At ∼34 weeks’ gestation* ^d^	0 (0–0)	0 (0–0)	0 (0–0)	0.0001	32	n/a
*At 6 months postpartum* ^e^	0 (0–0)	0 (0–0)	0 (0–0)	0.072	123	n/a
*At 12 months postpartum* ^c^	0 (0–0)	0 (0–0)	0 (0–0)	0.16	132	n/a
Hazardous drinking (defined as AUDIT consumption score ≥3)						
*At first antenatal visit, study enrolment* ^c^	147/870 (17%)	117/459 (25%)	30/411 (7%)	<0.0001	2	0
*At ∼34 weeks’ gestation* ^d^	43/840 (5%)	40/455 (9%)	3/385 (1%)	<0.0001	32	n/a
*At 6 months postpartum* ^e^	50/749 (7%)	40/403 (10%)	10/346 (3%)	<0.0001	123	n/a
*At 12 months postpartum* ^c^	92/740 (12%)	57/387 (15%)	35/353 (10%)	0.047	132	n/a
**World Health Organisation “Violence against Women” questionnaire measurements**						
Any intimate partner violence^f^						
*At first antenatal visit, study enrolment* ^c^	133/868 (15%)	101/457 (22%)	32/411 (8%)	<0.0001	4	0
*At 7 days’ (neonatal) visit* ^d^	41/871 (5%)	31/461 (7%)	10/410 (2%)	0.003	1	n/a
*At 12 months postpartum* ^c^	68/739 (9%)	40/388 (10%)	28/351 (8%)	0.27	133	16
**Edinburgh Postnatal Depression Scale (EPDS)**						
Total score						
*Enrolment (antenatal first booking)* ^g^	3 (1–7)	4 (1–8)	2 (1–6)	0.018	2	n/a
*At 6 weeks postpartum* ^g^	1 (0–4)	3 (0–5)	1 (0–2)	0.0001	51	n/a
*At 12 months postpartum* ^g^	1 (0–4)	2 (0–6)	0 (0–2)	0.0001	133	n/a
Probable depression: EPDS total score ≥13						
*Enrolment (antenatal first booking)* ^g^	75/870 (9%)	46/459 (10%)	29/411 (7%)	0.12	2	0
*At 6 weeks postpartum* ^g^	31/821 (4%)	19/435 (4%)	12/386 (3%)	0.34	51	n/a
*At 12 months postpartum* ^g^	43/739 (6%)	22/386 (6%)	21/353 (6%)	0.88	133	16
Possible depression: EPDS total score ≥10						
*Enrolment (antenatal first booking)* ^g^	145/870 (17%)	88/459 (19%)	57/411 (14%)	0.036	2	n/a
*At 6 weeks postpartum* ^g^	57/821 (7%)	38/435 (9%)	19/386 (5%)	0.032	51	n/a
*At 12 months postpartum* ^g^	65/739 (9%)	38/386 (10%)	27/353 (8%)	0.29	133	n/a
**Household food security at 12 months’ visit** ^h^				<0.0001	134	16
*No food insecurity*	461/738 (63%)	214/386 (55%)	247/352 (70%)			
*Has/is at risk of food insecurity*	277/738 (37%)	172/386 (45%)	105/352 (30%)			
**Combination indicator**				<0.0001	138	n/a
For hazardous drinking (enrolment), IPV (enrolment) and/or household food insecurity (12 months)						
*Has none of the above*	346/734 (47%)	133/382 (35%)	213/352 (61%)			
*Has one of the above*	283/734 (39%)	161/382 (42%)	122/352 (35%)			
*Has two of the above*	81/734 (11%)	67/382 (18%)	14/352 (4%)			
*Has all three of the above*	24/734 (3%)	21/382 (5%)	3/352 (1%)			

*Note*: Results are *n* (column %) with *p*‐value from chi^2^ test; mean (SD) with *p*‐value from *t*‐test for normally distributed variables; or median (interquartile range, IQR) with *p*‐value from Kruskal–Wallis.

Abbreviations: EPDS, Edinburgh Postnatal Depression Scale; HEU, HIV‐exposed uninfected; HU, HIV‐unexposed uninfected; SD, standard deviation.

^a^ Limited to mothers of children who tested HIV negative at the most recent test.

^b^ n/a, not applicable (no imputed datapoints used, as the variable was not included in the imputation or primary analysis models).

^c^With reference to the preceding 12 months.

^d^With reference to the time since mother identified her pregnancy.

^e^With reference to the time since birth of the infant.

^f^Any physical, sexual or psychological violence.

^g^With reference to the preceding week.

^h^Based on 10‐item questionnaire adapted from the Household Food Insecurity Access Scale (HFIAS), Food and Nutrition Technical Assistance Project (FANTA) and the Community Childhood Hunger Indentification Project Index (CCHIP).

### 3.2 Individual effects of potential maternal syndemic factors on child outcomes

Complete results from crude and adjusted regression models estimating individual effects of maternal HIV status, HD, IPV and HFIS on all pre‐specified child outcomes are shown in Tables S5–S9. The strongest relationships relevant to the current analysis were noted for absolute differences in WAZ; and relative differences in underweight, infectious admission hospitalisation in early infancy (7 days to 3 months), cognitive delay and motor delay (Table 3). Adjusting for HD, IPV and HFIS, maternal HIV predicted lower WAZ, increased risks of underweight, infectious hospitalisation and cognitive delay. In the same multivariable regression models, HD predicted lower WAZ and higher odds of underweight; IPV was associated with increased odds of motor delay; and HFIS with increased odds of cognitive and motor delay (Table 3).

**Table 3:** Estimates of absolute and relative differences in selected child growth, infectious morbidity and neurodevelopmental outcomes by maternal HIV status, hazardous drinking, IPV and household food security status

	WAZ Mean (SD)		Underweight (WAZ<−2)		Any infectious cause hospitalisation (age 7 days to 3 months)^a^		Composite cognitive score <85: delay^b^		Composite motor score <85: delay^b^	
Population outcome average/ prevalence	0.18 (1.18) over repeat measures		3% (155/4332 measurement events)		5% (40/849 with data)		7% (34/525 with assessments)		6% (32/510 with assessments)	
	β (95% CI)	aβ (95% CI)	OR (95% CI)	aOR (95% CI)	OR (95% CI)	aOR (95% CI)	OR (95% CI)	aOR (95% CI)	OR (95% CI)	aOR (95% CI)
HIV	−0.34 (−0.47; −0.21)	−0.28 (−0.41; −0.15)	2.73 (1.37; 5.44)	2.20 (1.09; 4.46)	3.30 (1.55; 7.02)	3.31 (1.52; 7.21)	2.19 (1.08; 4.44)	2.23 (1.06; 4.68)	2.01 (0.97; 4.12)	1.39 (0.64; 3.05)
Hazardous drinking	−0.41 (−0.59; −0.23)	−0.30 (−0.49; −0.10)	3.44 (1.57; 8.00)	2.53 (1.098; 5.93)	0.85 (0.35; 2.07)	0.47 (0.18; 1.28)	0.66 (0.23; 1.93)	0.46 (0.15; 1.43)	2.18 (0.97; 4.90)	1.35 (0.55; 3.32)
IPV	−0.23 (−0.41; −0.05)	−0.09 (−0.27; 0.10)	1.65 (0.70; 3.91)	1.04 (0.40; 2.65)	1.43 (0.64; 3.19)	1.33 (0.58; 3.06)	0.94 (0.32; 2.77)	0.88 (0.29; 2.72)	3.03 (1.33; 6.89)	2.35 (1.09; 4.90)
Food insecurity^c^	−0.11 (−0.24; 0.02)	−0.06 (−0.19; 0.06)	1.87 (0.91; 3.82)	1.50 (0.76; 2.95)	1.53 (0.78; 3.00)	1.34 (0.68; 2.67)	2.27 (1.13; 4.59)	2.09 (1.02; 4.29)	2.67 (1.29; 5.54)	2.31 (1.09; 4.90)

*Note*: Estimates from multi‐variable models are adjusted for maternal HIV status, hazardous drinking at enrolment, IPV at enrolment and household food insecurity at 12 months.

Abbreviations: aβ, adjusted mean difference from linear regression; aOR, adjusted odds ratio from logistic regression; CI, confidence interval; IPV, intimate partner violence; SD, standard deviation; WAZ, weight‐for‐age Z‐score.

^a^ Hospitalisation data obtained from electronic provincial hospital databases, infectious cause classification based on ICD10 codes and checked by infectious disease specialist paediatrician.

^b^ Composite score <85 per domain at approximately 12 months of age, using Bailey Scales of Infant Development, 3rd edition.

^c^ Has or is at risk of household food insecurity at 12 months, based on questionnaire adapted from the Household Food Insecurity Access Scale (HFIAS), Food and Nutrition Technical Assistance Project (FANTA) and the Community Childhood Hunger Indentification Project Index (CCHIP).

### 3.3 Combined effects of potential maternal syndemic factors on child outcomes

**Figure 2:**
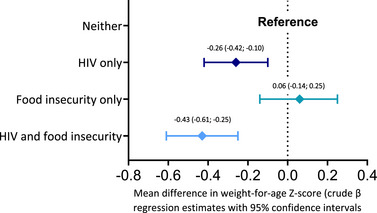
Mean differences in weight‐for‐age Z‐scores, by categories of maternal HIV status and household food security: interaction forest plots on linear scale.

**Figure 3:**
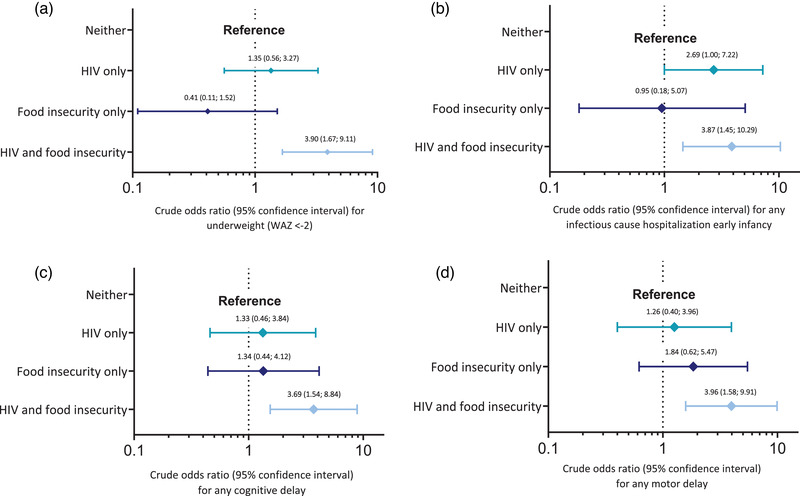
Relative odds (odds ratios) of child health outcomes, by categories of maternal HIV status and household food security: interaction forest plots on logarithmic scale. (a) Odds ratios for underweight (weight‐for‐age Z‐score < −2) during follow‐up. (b) Odds ratios for infectious cause hospitalisation between ages 7 days and 3 months. (c) Odds ratios for any cognitive delay (Bayley Scales of Infant Development, 3rd edition, composite score). (d) Odds ratios for any motor delay (Bayley Scales of Infant Development, 3rd edition, composite score).

Epidemiologic interaction estimates (IC and RERI_RR_) are shown in Table S10. Across child health outcomes, the strongest evidence for positive interaction effects was for *maternal HIV status* (exposed, HIV+ vs. unexposed, HIV−) and *household food insecurity (HFIS)* (HFIS+ vs. HFIS−); these effects were most evident for WAZ, underweight, infectious hospitalisation and developmental delay (Table 4 and Tables S5–S9). For example, using food‐secure CHU (HIV−|HFIS−) as reference, food‐secure CHEU (HIV+|HFIS−) had WAZ −0.26 (95% CI −0.42 to −0.10) and CHEU with food insecurity (HIV+|HFIS+) had WAZ −0.43 (95% CI −0.61; −0.25), whereas CHU with food insecurity (HIV−|HFIS+) had a similar WAZ to the reference (HIV−|HFIS+), WAZ 0.06 (95% CI −0.14; 0.25), Figure 2 and Table 4. That is, whereas exposure to only HFIS was not associated with WAZ deficit (mean difference 0.06), exposure to only HIV was associated with some WAZ deficit (−0.26), and double exposure (HFIS and HIV) was associated with a substantial WAZ deficit (−0.43), which was greater than the sum of the effects of the individual exposures (−0.26 + 0.06 = −0.20, compared to −0.43) [27]. Similar results were seen for the odds of being underweight (Figure 3a), infectious cause hospitalisation in early infancy (Figure 3b), cognitive delay (Figure 3c) and motor delay (Figure 3d); (Table 4 and Tables S5–S9). There were also suggestions of positive HIV‐HFIS interactions on HCAZ, microcephaly and hospitalisation beyond the first 72 hours, although precision was limited (Tables S4–S9).

**Table 4:** Summarised comparison of child growth, infectious morbidity and neurodevelopmental outcomes by maternal HIV status and household food security status: estimates and measures of interaction effects

	WAZ Mean (SD)		Underweight (WAZ<−2)		Any infectious cause hospitalisation (age 7 days to 3 months)^a^		Composite cognitive score <85: delay^b^		Composite motor score <85: delay^b^	
Study population outcome average/ prevalence	0.18 (1.18) over repeat measures		3% (155/4332 measurement events)		5% (40/849 with data)		7% (34/525 with assessments)		6% (32/510 with assessments)	
Regression coefficients	β (95% CI)	*p*	OR (95% CI)	*p*	OR (95% CI)	*p*	OR (95% CI)	*p*	OR (95% CI)	*p*
Categories of exposure to HIV and food security										
*No HIV, food secure*	Ref		Ref		Ref		Ref		Ref	
*HIV, food secure*	−0.26 (−0.42; −0.10)	0.001	1.35 (0.56; 3.27)	0.51	2.69 (1.00; 7.22)	0.05	1.33 (0.46; 3.84)	0.60	1.26 (0.40; 3.96)	0.69
*No HIV, food insecure*	0.06 (−0.14; 0.25)	0.55	0.41 (0.11; 1.52)	0.18	0.95 (0.18; 5.07)	0.95	1.34 (0.44; 4.12)	0.61	1.84 (0.62; 5.47)	0.27
*HIV and food insecure*	−0.43 (−0.61; −0.25)	<0.0001	3.90 (1.67; 9.11)	0.002	3.87 (1.45; 10.29)	0.007	3.69 (1.54; 8.84)	0.003	3.96 (1.58; 9.91)	0.003
Interaction model^c^										
*HIV versus no HIV*	−0.26 (−0.42; −0.10)	0.001	1.35 (0.56; 3.27)	0.51	2.69 (1.00; 7.22)	0.05	1.33 (0.46; 3.83)	0.60	1.26 (0.40; 3.96)	0.69
*Food insecure versus secure, no HIV*	0.06 (−0.15; 0.25)	0.55	0.41 (0.11; 1.52)	0.18	0.95 (0.18; 5.07)	0.94	1.34 (0.44; 4.12)	0.61	1.84 (0.62; 5.47)	0.27
*Interaction term*	−0.23 (−0.48; 0.02)	0.068	7.05 (1.43; 34.70)	0.16	1.52 (0.24; 9.60)	0.65	2.07 (0.46; 9.29)	0.34	1.70 (0.37; 7.84)	0.50
Interaction contrast (IC)^d^	n/a		12%		2%		−6%		6%	
RERI_RR_ ^e^	n/a		1.49		1.10		−1.33		1.54	
Multiplicative interaction measure^f^	n/a		3.68		1.38		0.19		1.57	
Evidence of interaction on additive scale	Yes, positive		Yes, positive		Yes, positive		Yes, negative		Yes, positive	
Evidence of interaction on multiplicative scale	n/a		Yes, positive		Yes, positive		Yes, positive		Yes, positive	

*Note*: Proportions are expressed as percentages to facilitate interpretation in clinical terms; epidemiological measures of interaction only calculable on binary data. Has or is at risk of household food insecurity, based on questionnaire adapted from the Household Food Insecurity Access Scale (HFIAS), Food and Nutrition Technical Assistance Project (FANTA) and the Community Childhood Hunger Indentification Project Index (CCHIP).

Abbreviations: CI, confidence interval; n/a, not applicable; OR, odds ratio; RERI_RR_, relative excess risk due to interaction, also referred to as interaction contrast ratio (ICR); SD, standard deviation; WAZ, weight‐for‐age Z‐score.

^a^ Hospitalisation data obtained from electronic provincial hospital databases, infectious cause classification based on ICD10 codes and checked by infectious disease specialist paediatrician.

^b^ Composite score <85 per domain at approximately 12 months of age, using Bailey Scales of Infant Development, 3rd edition.

^c^Models including only HIV and binary indicator for household food insecurity, with interaction term (Z‐scores analysis accounting for repeat measures; model choice based on data type).

^d^Indicator for the differences in proportions between the four exposure groups (formula, p_11_ − p_10_ − p_01_ + p_00_, where p_11_ indicates risk of binary outcome in group exposed to both HIV and food insecurity; p_10_, exposure to HIV only; p_01_, exposure to food insecurity only; and p_00_, exposed to neither). Measures interaction on the additive scale: >0 indicates positive interaction, <0 indicates negative interaction. Interpretable as magnitude of interaction on additive scale (absolute differences in risk); can be approximated by the difference in mean Z‐scores indicated by interaction term in linear regression model.

^e^RERI_RR_ tests interaction on the additive scale, based on calculated risk ratios; interpretation: <0, negative interaction; RERI_RR_>0, positive interaction (RERI_RR_ measures departure from additive effects, in terms of direction but not magnitude as baseline group risks may vary between groups).

^f^Measures the extent to which, on the risk ratio scale, the effect of both exposures together exceeds the product of the effects of the two exposures considered separately; if <1, negative interaction; >1, positive; can be approximated by the interaction term of logistic regression if rare disease assumption holds (here, OR in interaction terms are likely an overestimation).

Precision was also limited for subgroup comparisons by exposure categories for *maternal HIV status* (HIV+ vs. HIV−) and *hazardous drinking* (HD+ vs. HD−, as reported at study enrolment). The concurrent exposure of HD exacerbated the adverse effects of HIV exposure on mean weight (Table S5): compared to children exposed to neither (HIV−|HD−), those exposed to HIV only (HIV+|HD−) had WAZ −0.28 (95% CI −0.41; −0.14) and HD only (HIV−|HD+) had WAZ −0.28 (95% CI −0.65; 0.08), while those with both exposures (HIV+|HD+) had WAZ −0.61 (95% CI −0.81; −0.40). There was also evidence of interaction between exposures to HIV and HD on the odds of underweight when comparing HIV exposure only (HIV+|HD−), HD exposure only (HIV−|HD+) and both exposures (HIV+|HD+) to neither exposure (HIV−|HD−): odds ratio, OR (95% CI) of 2.31 (1.11; 4.82), 3.57 (CI 0.84; 15.13) and 6.01 (2.22; 16.22), respectively (Tables S6 and S10). Similar results were seen for LAZ and ambulatory diarrhoea, but not for other measures of infectious morbidity or for developmental delay (Tables S5–S7).

Exposure to both *HIV and IPV* predicted substantially higher odds of ambulatory childhood illness and motor delay than either exposure by itself (positive interaction), albeit with low precision (Tables S7 and S 9). Compared to children with neither exposure (HIV−|IPV−), those with HIV exposure only (HIV+|IPV−) had double the odds of RTI (OR 2.53; 95% CI 1.64; 3.90); those with IPV exposure only (HIV−|IPV+) had similar odds (OR 0.32; 95% CI 0.04; 2.39); and those with both exposures (HIV+|IPV+) had three‐fold higher odds (OR 3.27; 95% CI 1.85; 5.78). Results were similar for ambulatory diarrhoea (Table S7). Compared to neither exposure (HIV−|IPV−), odds for motor delay was incrementally increased for HIV exposure only (HIV+|IPV−), IPV exposure only (HIV−|IPV+) and both exposures (HIV+|IPV+) with OR (95% CI) of 1.61 (0.70; 3.75), 2.14 (0.45; 10.21) and 4.68 (1.72; 12.72), respectively.

### 3.4 Sensitivity analyses

Results from complete‐case analysis using pre‐imputation data approximated those from analyses based on imputed datasets. Limiting all outcomes and exposures to the 12‐month time point did not alter any inferences. Results were also unchanged when varying the choice of time point for measurements of exposure to maternal alcohol use, depression or IPV (data not shown).

## 4 DISCUSSION

We provide unique insights into the syndemic vulnerability from HD, IPV and HFIS in WLHIV and their HIV‐negative children, with data to support all three criteria of a syndemic: (a) adverse contextual and social factors; (b) disease clustering; and (c) adverse disease interaction [11]. We described a population of women and children living in adverse socio‐economic conditions, within a country known for its stark social inequality, a legacy of extreme racism and ongoing structural violence [32]. Notably, HFIS was the most prevalent maternal syndemic co‐factor for both WLHIV and HIV‐negative women, with many households experiencing hunger [31]. WLHIV also had greater risks of other adverse exposures than HIV‐negative women across socio‐economic measures. Within this context, there was marked clustering of maternal HIV with HD, IPV and HFIS at enrolment and over time, with evidence of bi‐directional relationships. In turn, these factors individually affected child health outcomes, in both crude and adjusted analyses. Moreover, we found evidence for interaction with HIV for each of the examined syndemic co‐factors, on one or more of the examined child outcomes. The strongest interaction effects were seen between maternal HIV and HFIS. Taken together, these data support our *a priori* hypotheses, suggesting a maternal HIV, alcohol, violence and food insecurity syndemic with adverse consequences for CHEU health.

Although concurrence of and interactions between the maternal factors included in this analysis have been reported in several adult‐focused studies [14, 33], the impact on child health is less well understood. To our knowledge, this is the first study to systematically seek evidence of HIV‐related syndemic interactions on a maternal level, with impact measured on a child health level, following current syndemic theory [11]. Cross‐generational syndemic vulnerability has been described in non‐HIV‐related work, demonstrating the complex web of interactions that can persist over generations, driving psychological and cardiometabolic disease [34]. While our findings require corroboration in other HIV‐specific settings, these results align with growing evidence for the developmental origins of the health and disease hypothesis, strengthened by recent advances in neuro‐imaging and epigenetic research [35, 36].

These data demonstrate a clear need for multi‐faceted interventions to optimise child health in high HIV burden settings, in addition to breastfeeding with ART for maternal HIV. There are a growing number of adult‐focused studies evaluating individual‐ and community‐level interventions to reduce alcohol abuse, IPV and/or food insecurity. Community mobilisation, screening with counselling, motivational interviews, housing interventions, cognitive behaviour therapy and microfinance interventions have proven efficacy, although results have varied by setting and duration of effect [33, 37, 38]. In addition to policy‐level decision‐making around water security and agricultural practices, household‐level interventions, including home gardens, food rations and other economic strengthening approaches, can mitigate food insecurity [31, 39, 40, 41]. However, there are limited published data available on the effects similar single or multi‐component interventions may have on the health of CHEU. The South African‐based Philani Intervention Programme, utilising a “Mentor‐Mother” community field worker approach, has demonstrated improvements in maternal mental health and child cognitive and physical growth [42]. The feasibility and success of this programme highlights a potential avenue for implementation of and linkage to additional interventions for HD, IPV and HFIS in a similar context. In turn, such multi‐faceted programmes would align with the Sustainable Development Goals, which seek to bring “Health in All Policies” [43].

Several key exposures evaluated in this analysis were measured with self‐report, increasing the risk of information bias, even when utilising validated tools [27]. Reassuringly, as the same measurement tools were used for both groups of women, any resulting bias would tend towards the null [27]. The food insecurity findings would have been strengthened by repeated measurement, and the use of more than one tool. Our study is also limited by the small sub‐group sample sizes, most notably the low prevalence of probable maternal depression overall, and of HD in HIV‐negative women. Further research into this field should ideally explore maternal mental health effects in greater detail while incorporating larger sample sizes, for which purpose individual‐patient‐data meta‐analysis may prove useful. Furthermore, a full appreciation of syndemic effects requires both individual‐ and population‐level data [11]. Our findings may not be generalisable to settings with different socio‐economic and political structures. Changes in HIV treatment policies have occurred since the completion of our study, most notably the shift from efavirenz‐based to dolutegravir‐based ART for pregnant women. In turn, the health trajectories of CHEU in our study cohort may not be generalisable to CHEU whose mothers received dolutegravir in pregnancy. Nonetheless, it is unlikely that the adverse consequences of exposure to maternal psychosocial/behavioural factors would differ meaningfully by the type of antenatal ART regimen. Additionally, the background estimates of maternal alcohol use, IPV and HFIS have increased substantially since the COVID‐19 pandemic, including in South Africa [44]. Given the worsening international economic milieu, it is likely that our (pre‐COVID‐19) results underestimate the current syndemic vulnerability of HIV‐affected families.

Of the syndemic co‐factors evaluated in this work, the most time‐critical and salient aspect requiring urgent attention is the extreme burden of food insecurity. There has been a devastating post‐2020 increase in food insecurity globally, with worsening conditions predicted under current rates of climate change [31]. Africa—where most CHEU live—has been identified as the continent with the greatest risk of hunger for the present and the foreseeable future [31]. Optimising CHEU health cannot be achieved without addressing the dire living conditions many HIV‐affected families experience daily, including the enduring spectre of child hunger.

## 5 CONCLUSIONS

Syndemic interactions between maternal HIV, HD, IPV and HFIS may partly drive the residual health discrepancies between CHEU and CHU. These data support the importance of holistic maternal and family care and support alongside ART to optimise CHEU child health.

## COMPETING INTERESTS

The authors have no competing interests to declare.

## AUTHOR CONTRIBUTIONS

SMLR (corresponding author) was responsible for the conceptualisation and implementation of the HU2 study; assisted with the collection of data; conducted the analyses; wrote the first draft of the manuscript and confirms that she had full access to all the data and takes final responsibility for the decision to submit for publication. LM and EJA conceived the MCH‐ART and HU2 studies, and were responsible for study design, funding, implementation and overall leadership. TKP was the study coordinator for the MCH‐ART study and was responsible for data management and oversight. AZ was the senior MCH‐ART study manager and provided oversight of all study administration processes. All authors contributed to and approved the final manuscript.

## FUNDING

Funding for this study was provided by PEPFAR through NICHD under Cooperative Agreement 1R01HD074558. Additional funding comes from the Elizabeth Glaser Pediatric AIDS Foundation, South African Medical Research Council (Clinician‐Researcher PhD Scholarship), the Fogarty Foundation (NIH Fogarty International Center Grant #5R25TW009340) and the Office of AIDS Research. TKP was supported by the Fogarty International Center of the National Institutes of Health under Award Number K43TW011943.

## Supporting information


**Figure S1**: Schematic representation of hypothesised maternal HIV‐related syndemics and pathways to adverse child health consequences.
**Figure S2**: Study flow diagram.
**Figure S3**: a) Proportional Venn diagram demonstrating overlap between maternal HIV status, hazardous drinking, and intimate partner violence; b). Proportional Venn diagram demonstrating overlap between maternal HIV status, hazardous drinking, and household food insecurity; c) Proportional Venn diagram demonstrating overlap between maternal HIV status, intimate partner violence, and household food insecurity.
**Table S1**: Sample size calculations1 for primary study analyses on child health outcomes.
**Table S2**: Characteristics of mother‐infant pairs who completed follow‐up and contributed household food insecurity data at 12 months' study visit vs those who did not, stratified by maternal HIV status.
**Table S3**: Methodological aspects of multiple imputation overall and by outcome.
**Table S4**: Interrelationships between maternal socio‐demographic and behavioural factors: percentage of total study population with two potentially adverse maternal or household factors.
**Table S5**: Mean differences in Z‐scores by maternal characteristics: results from random effects linear regression models with repeat measures.
**Table S6**: Relative odds (odds ratios) for underweight, stunting, and microcephaly over time, by maternal characteristics: results from random effects logistic regression models with repeat measures.
**Table S7**: Relative odds (odds ratios) for infectious morbidity events, by maternal characteristics: results from logistic regression models.
**Table S8**: Mean differences in BSID‐III composite developmental scores, by maternal characteristics, at approximately 12 months of age: results from linear regression models.
**Table S9**: Relative odds (odds ratios) of developmental delay (BSID‐III composite score <85), by maternal characteristics, at approximately 12 months of age: results from logistic regression models.
**Table S10**: Interaction on the additive and/or the multiplicative scale: testing variation in exposure effects by strata of maternal HIV status, hazardous drinking, intimate partner violence, and household food insecurity, using crude risk ratios and epidemiological measures of interaction.

## Data Availability

The data that support the findings of this study are available from the corresponding author upon reasonable request.
