# Supplementary material for: Children of a syndemic: co‐occurring and mutually reinforcing adverse child health exposures in a prospective cohort of HIV‐affected mother‐infant dyads in Cape Town, South Africa
Source: J Int AIDS Soc. 2023 Nov 1;26(Suppl 4):e26152. doi: 10.1002/jia2.26152 (PMC10618899; doi:10.1002/jia2.26152)
Supplement: Supplementary file 1 — Figure S1: Schematic representation of hypothesised maternal HIV‐related syndemics and pathways to adverse child health consequences. Figure S2: Study flow diagram. Figure S3: a) Proportional Venn diagram demonstrating overlap between maternal HIV status, hazardous drinking, and intimate partner violence; b). Proportional Venn diagram demonstrating overlap between maternal HIV status, hazardous drinking, and household food insecurity; c) Proportional Venn diagram demonstrating overlap between maternal HIV status, intimate partner violence, and household food insecurity. Table S1: Sample size calculations1 for primary study analyses on child health outcomes. Table S2: Characteristics of mother‐infant pairs who completed follow‐up and contributed household food insecurity data at 12 months' study visit vs those who did not, stratified by maternal HIV status. Table S3: Methodological aspects of multiple imputation overall and by outcome. Table S4: Interrelationships between maternal socio‐demographic and behavioural factors: percentage of total study population with two potentially adverse maternal or household factors. Table S5: Mean differences in Z‐scores by maternal characteristics: results from random effects linear regression models with repeat measures. Table S6: Relative odds (odds ratios) for underweight, stunting, and microcephaly over time, by maternal characteristics: results from random effects logistic regression models with repeat measures. Table S7: Relative odds (odds ratios) for infectious morbidity events, by maternal characteristics: results from logistic regression models. Table S8: Mean differences in BSID‐III composite developmental scores, by maternal characteristics, at approximately 12 months of age: results from linear regression models. Table S9: Relative odds (odds ratios) of developmental delay (BSID‐III composite score <85), by maternal characteristics, at approximately 12 months of age: results from logistic regression models. Table S10: [file JIA2-26-e26152-s001.pdf]

APPENDIX

Table A1. Sample size calculations[a] for primary study analyses on child health outcomes

Outcome	Expected distributional values	Minimum detectable difference[b]	Sample size required[c]	Estimated power
Absolute difference in mean Z-scores	SD = 1.0	RD: 0.2 of a Z-score	880 (achieved)	>80%
Absolute difference in proportions of children with diarrhoeal illness	Underlying probability of diarrhoeal risk in HIV-unexposed infants, p = 0.15	RD: 0.08	880 (achieved)	>80%
Relative difference in proportions of children with diarrhoeal illness	Underlying probability of diarrhoeal risk in HIV-unexposed infants, p = 0.15	RR: 1.5	880 (achieved)	>80%
Absolute difference in mean composite score for developmental domains (BSID-III)	SD = 15 points	RD: 5 points	500 (achieved)	>90%

Abbreviations: BSID-III, Bayley Scales of Infant Development, 3rd edition; RD, absolute risk difference; RR, relative risk; SD, standard deviation.
[a]All sample size estimations calculated specifically for postnatal comparison of children who were HIV-exposed uninfected compared to HIV unexposed, *a priori* with two-sided alpha of 0.05.
[b]Based on clinically meaningful differences defined *a priori* following independent expert opinions and previous literature: for growth, >|0.2| of a Z-score; for morbidity, >|0.08| absolute and 50% relative differences (RR>1.5 or <0.5); for neurodevelopment, >|5| points on the composite scale in absolute difference, or >20% relative difference (RR>1.2 or <0.8).
[c]Including anticipated early loss-to-follow-up and HIV transmission.

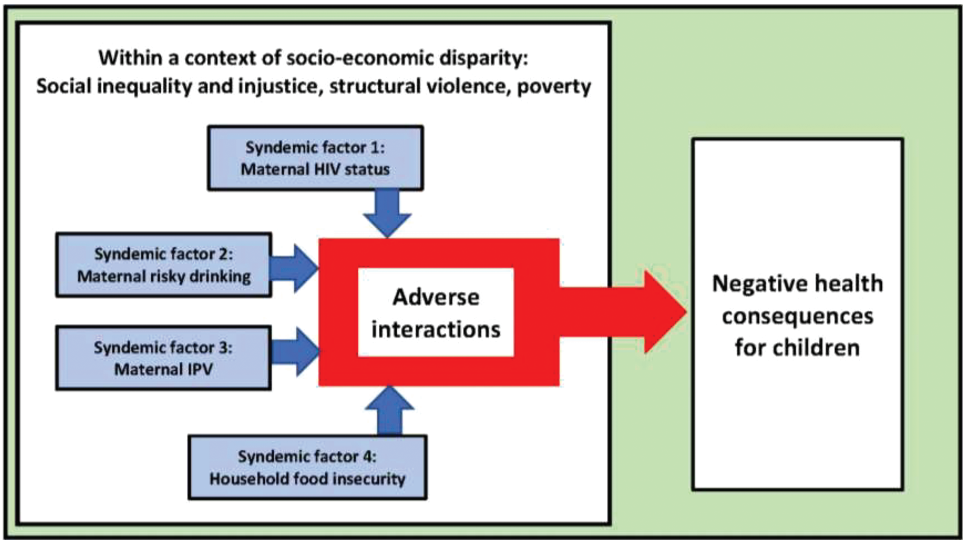

Figure A1. Schematic representation of hypothesized maternal HIV-related syndemics and pathways to adverse child health consequences.
Abbreviation: IPV, intimate partner violence.

Table A2. Characteristics of mother-infant pairs who completed follow-up and contributed household food insecurity data at 12 months' study visit versus those who did not, stratified by maternal HIV status

	Women who are HIV positive, and CHEU				Women who are HIV negative, and CHU			
**Characteristic**	**Total $N = 461$**	**Food security data missing $n = 75$**	**Food security data available $n = 386$**	***p***	**Total $N = 411$**	**Food security data missing $n = 59$**	**Food security data available $n = 352$**	***p***
**Demographics and household characteristics at study enrolment**								
Age in years, mean (SD)	29 (5)	27 (5)	29 (6)	0.0006	28 (6)	26 (6)	28 (6)	0.02
Married/cohabiting	189 (41%)	30 (40%)	159 (41%)	0.85	184 (45%)	22 (37%)	162 (46%)	0.21
Incomplete secondary education	347 (75%)	54 (72%)	293 (76%)	0.47	227 (55%)	30 (51%)	197 (56%)	0.46
Unemployed	279 (60%)	39 (52%)	240 (62%)	0.10	217 (53%)	30 (51%)	187 (53%)	0.75
Household crowding (≥10 people)	28 (6%)	4 (5%)	24 (6%)	0.77	12 (3%)	0	12 (3%)	0.15
Informal housing	242(52%)	43 (57%)	199 (52%)	0.36	196 (48%)	25 (42%)	171 (48%)	0.38
Lives in a formal, brick home, with flushable toilet and running water inside the home				0.85				0.17
No, lacks at least one of the above	336 (73%)	54 (72%)	282 (73%)		256 (62%)	32 (54%)	224 (64%)	
Yes, has all three	125 (27%)	21 (28%)	104 (27%)		155 (38%)	27 (46%)	128 (36%)	
**Maternal HIV-related measures at enrolment and delivery**								
CD4 cell count at ART initiation (100 cells/mm^3)	4.02 (2.11)	4.34 (2.13)	3.95 (2.10)	0.11	–	–	–	
Log_{10} HIV viral load at ART initiation (copies/ml)	3.86 (0.96)	3.64 (0.97)	3.91 (0.95)	0.02	–	–	–	
Log_{10} HIV viral load at delivery (copies/ml)	1.84 (0.63)	1.96 (0.80)	1.82 (0.59)	0.20	–	–	–	
HIV viral load <50 copies/ml at delivery	352 (76%)	55 (73%)	297 (77%)	0.50	–	–	–	

(*Continued*)

Table A2. (Continued)

Characteristic	Women who are HIV positive, and CHEU				Women who are HIV negative, and CHU			
	Total $N = 461$	Food security data missing $n = 75$	Food security data available $n = 386$	p	Total $N = 411$	Food security data missing $n = 59$	Food security data available $n = 352$	p
**Infant characteristics**								
Preterm at birth (<37 weeks)	56 (12%)	8 (11%)	48 (12%)	0.67	38 (9%)	3 (5%)	35 (10%)	0.23
Small-for-gestational-age (birthweight <10th centile)[a]	51 (11%)	2 (3%)	49 (13%)	0.01	39 (9%)	7 (12%)	32 (9%)	0.50
Male sex	232 (50%)	40 (53%)	192 (50%)	0.57	196 (48%)	33 (56%)	163 (46%)	0.17
**Psychosocial and behavioural factors, peripartum**								
Hazardous drinking (AUDIT-C ≥3) at study enrolment[b]	117/459 (25%)	15/75 (20%)	102/384 (27%)	0.23	30 (7%)	3 (5%)	27 (8%)	0.48
Hazardous drinking (AUDIT-C ≥3) during known pregnancy	40/455 (9%)	5/74 (7%)	35/381 (9%)	0.50	3/385 (1%)	1/55 (2%)	2/330 (1%)	0.34
Probable depression at study enrolment (EPDS ≥13)[c]	46/459 (10%)	8/75 (11%)	38/384 (10%)	0.84	29 (7%)	4 (7%)	25 (7%)	0.93
Probable depression at 6 weeks' postpartum visit (EPDS ≥13)[c]	19/435 (4%)	4/63 (6%)	15/372 (4%)	0.41	12/386 (3%)	2/47 (4%)	10/339 (3%)	0.63
Any intimate partner violence at study enrolment[a]	101/457 (22%)	14/75 (19%)	87/382 (23%)	0.43	32 (8%)	5 (8%)	27 (8%)	0.83
Any intimate partner violence during pregnancy[a]	31 (7%)	5 (7%)	26 (7%)	0.98	10 (2%)	0	10 (3%)	0.19

Note: Results are *n*(column %) with *p*-value from chi^2 test; mean (sd) with *p*-value from *t*-test for normally distributed variables; or median (interquartile range, IQR) with *p*-value from Kruskal-Wallis for non-normally distributed variables.

Abbreviations: ART, antiretroviral therapy; AUDIT, Alcohol Use Disorder Identification Tool; AUDIT-C, AUDIT consumption score; CHEU, children who are perinatally HIV exposed but HIV negative; CHU, children who are not perinatally HIV exposed and are HIV negative; EPDS, Edinburgh Postnatal Depression Scale; ml, millilitre; mm^3, cubic millimetre; SD, standard deviation.

[a]Any physical, sexual or psychological violence in the last 12 months, based on the World Health Organization violence against women questionnaire.

[b]Hazardous drinking, defined as Alcohol use disorders identification tool consumption (AUDIT-C) score ≥3, with reference to the previous 12 months.

[c]EPDS total score and threshold measures refer to "the last week."

Table A3. Methodological aspects of multiple imputation overall and by outcome

**OVERALL**	
Method	A multiple imputation by chained equations (MICE) approach was used, assuming missing values to be missing at random. This approach, also known as sequential regression multivariate imputation, was chosen to accommodate the different distributional forms of key variables. We used the **mi impute chained** command in Stata 16.1, Statistical Software: StataCorp LLC.
Principles	Twenty datasets were imputed, with regression estimates combined using Rubin's rules. Imputed datapoints were excluded from analysis for (1) missing *outcome* data and (2) missing predictor variables that were measurable beyond the date of infant or maternal demise. All imputations were stratified by study to accommodate interaction testing in analytic models and maintain compatibility between imputation and analytic models.
Imputation model choices	Binary variables were imputed using augmented logistic regression, and continuous variables using linear regression.
Model checking	Conducted as relevant to primary imputation models. Observed and imputed mean data points and distributions checked for similarity and sensibility of values post-imputation.
**PER OUTCOME**	
**Growth**	Infant anthropometric measures over time
Model choices	Continuous outcome variables were imputed in a single model (Z-scores for weight-for-age, WAZ; length-for-age, LAZ; head circumference-for-age, HCAZ; and weight-for-length, WLZ). Binary outcomes were imputed in a separate, single model (underweight, stunting, microcephaly and wasting defined as relevant Z-score<-2 following international standard definitions).
Imputed variables	1. All Z-scores (as above), with intimate partner violence at enrolment; intimate partner violence at 12 months; probable depression at enrolment; probable depression at 12 months postpartum; hazardous drinking at enrolment; hazardous drinking at 12 months postpartum; binary indicators for household food insecurity and for early initiation of breastfeeding.<br>2. Binary indicators for underweight, stunting, microcephaly and wasting; intimate partner violence at enrolment; intimate partner violence at 12 months; probable depression at enrolment; probable depression at 12 months postpartum; hazardous drinking at enrolment; hazardous drinking at 12 months postpartum; binary indicators for household food insecurity and for early initiation of breastfeeding.
Regular variables	Continuous: gestation at birth (weeks); weight-for-age Z-score at birth; breastfeeding duration (months)<br>Binary: maternal employment (any vs. none); education (secondary school completed vs. not); housing (formal housing vs. not); marital status (married/co-habiting vs. single); household crowding (>10 people vs. not); presence of amenities (flush toilet in home, running water in home)
**Infectious morbidity**	Hospitalisation and ambulatory childhood illness (presumed lower respiratory tract infection; diarrhoeal illness)
Model choices	All outcomes modelled as binary variables using a single logistic imputation model.
Imputed variables	Binary outcome variables (all yes vs. no):<br>1. Ever hospitalised<br>2. Ever hospitalised, after the first 72 hours<br>3. Any infectious cause hospitalisation between 7 days and 3 months of age<br>4. Ever reported to have presumed lower respiratory tract infection at a study visit<br>5. Ever reported to have diarrhoeal illness at a study visit<br>Predictor variables:Intimate partner violence at enrolment; intimate partner violence at 12 months; probable depression at enrolment; hazardous drinking at enrolment; hazardous drinking at 12 months postpartum; binary indicators for household food insecurity, for completed childhood vaccination status (at age of 3 months) and for early initiation of breastfeeding.
Regular variables	Continuous: gestation at birth (weeks); weight-for-age Z-score at birth; breastfeeding duration (months)<br>Binary: maternal employment (any vs. none); education (secondary school completed vs. not); housing (formal housing vs. not); marital status (married/co-habiting vs. single); household crowding (>10 people vs. not); presence of amenities (flush toilet in home, running water in home)

(*Continued*)

Table A3. (Continued)

PER OUTCOME	
**Neurodevelopment**	Bayley Scales of Infant Development (BSID-III) at 12 months' developmental visit
Model choices	Composite scores for cognitive, motor and language scores were imputed using a single linear regression model. Binary indicators for delay (BSID-III composite score < 85) were imputed using a single logistic regression model.
Imputed variables	1. Composite cognitive score; composite motor score; composite language score; intimate partner violence at enrolment; intimate partner violence at 12 months; probable depression at enrolment; probable depression at 12 months postpartum; hazardous drinking at enrolment; hazardous drinking at 12 months postpartum; binary indicators for household food insecurity and for early initiation of breastfeeding.<br>2. Binary indicators for cognitive delay, motor delay, language delay; intimate partner violence at enrolment; intimate partner violence at 12 months; probable depression at enrolment; probable depression at 12 months postpartum; hazardous drinking at enrolment; hazardous drinking at 12 months postpartum; binary indicators for household food insecurity and for early initiation of breastfeeding.
Regular variables	Continuous: gestation at birth (weeks); weight-for-age Z-score at birth; breastfeeding duration (months) Binary: maternal employment (any vs. none); education (secondary school completed vs. not); housing (formal housing vs. not); marital status (married/co-habiting vs. single); household crowding (>10 people vs. not); presence of amenities (flush toilet in home, running water in home)

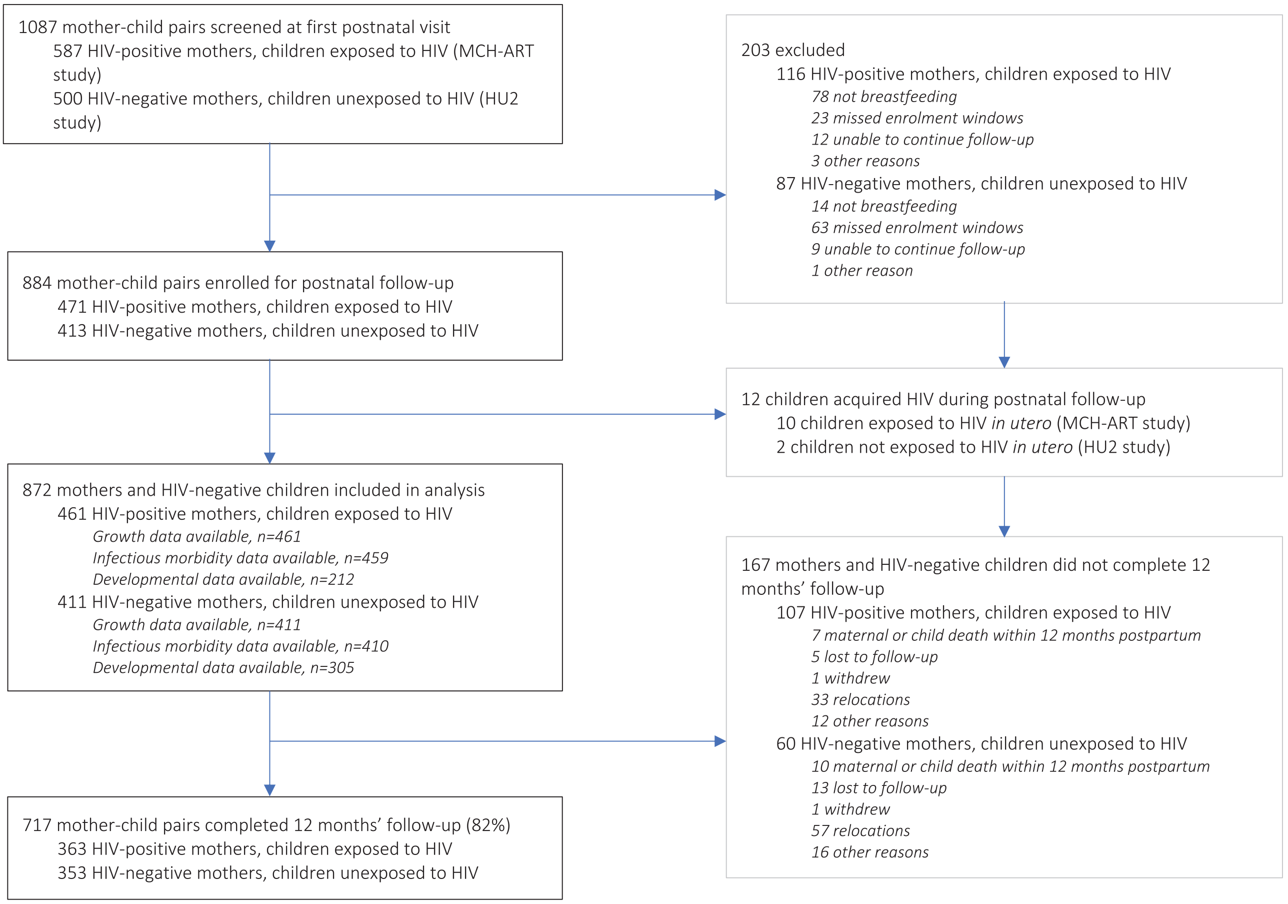

Figure A2. Study flow diagram.

Table A4. Inter-relationships between maternal socio-demographic and behavioural factors: percentage of total study population with two potentially adverse maternal or household factors

	Food insecurity[a]	Household crowding	No running water	Not matriculated	IPV[b]	Hazardous drinking[c]	HIV
**HIV**	23% (172/738)	3% (28/872)	31% (272/872)	40% (347/872)	12% (101/868)	13% (117/870)	**TOTAL in population 53% (461/872)**
**Hazardous drinking**	8% (58/736)	2% (14/870)	8% (71/870)	11% (99/870)	6% (50/868)	**TOTAL in population 17% (147/870)**	13% (117/870)
**IPV**	7% (51/734)	1% (9/868)	8% (72/868)	11% (93/868)	**TOTAL in population 15% (133/868)**	6% (50/868)	12% (101/868)
**Not matric-ulated**	27% (200/738)	4% (31/872)	40% (345/872)	**TOTAL in population 66% (574/872)**	11% (93/868)	11% (99/870)	40% (347/872)
**No running water**	20% (147/738)	1% (6/872)	**TOTAL in population 54% (468/872)**	40% (345/872)	8% (72/868)	8% (71/870)	31% (272/872)
**Household crowding**	2% (18/738)	**TOTAL in population 5% (40/872)**	1% (6/872)	4% (31/872)	1% (9/868)	2% (14/870)	3% (28/872)
**Food insecurity**	**TOTAL in population 38% (277/738)**	2% (18/738)	20% (147/738)	27% (200/738)	7% (51/734)	8% (58/736)	23% (172/738)

Note: Data availability: socio-demographics and HIV status, n = 872; intimate partner violence, n = 868; hazardous drinking, n = 870; household food insecurity, only measured at 12 months' visit, n = 738.
Abbreviation: IPV, intimate partner violence.
[a]Household has or is at risk of food insecurity, based on questionnaire adapted from the Household Food Insecurity Access Scale (HFIAS), Food and Nutrition Technical Assistance Project (FANTA) and the Community Childhood Hunger Indentification Project Index (CCHIP).
[b]Any physical, sexual or psychological violence as measured with World Health Organization violence against women questionnaire.
[c]Hazardous drinking, defined as Alcohol use disorders identification test (AUDIT-C) score ≥3.

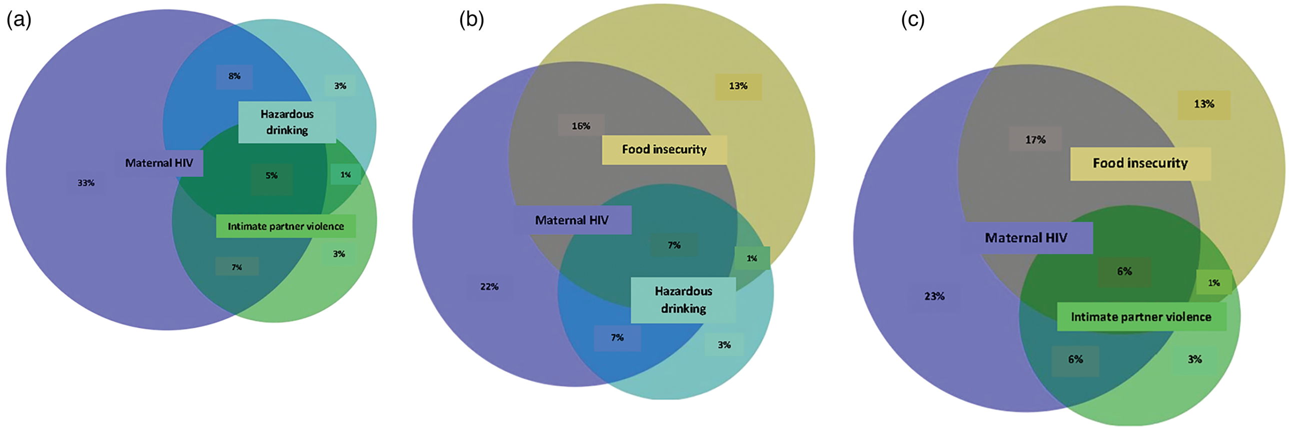

Figure A3. (a) Proportional Venn diagram demonstrating the overlap between maternal HIV status, hazardous drinking and intimate partner violence.
Household food insecurity as measured at 12 months' postpartum visit using questionnaire adapted from the Household Food Insecurity Access Scale (HFIAS), Food and Nutrition Technical Assistance Project (FANTA) and the Community Childhood Hunger Indentification Project Index (CCHIP); IPV, intimate partner violence: any physical, sexual or psychological violence (WHO VAW questionnaire) as reported at study enrolment.
***N* = 734 with data on all three variables; 31% have no HIV, IPV or food insecurity.**
(b) Proportional Venn diagram demonstrating the overlap between maternal HIV status, hazardous drinking and household food insecurity.
(c) Proportional Venn diagram demonstrating the overlap between maternal HIV status, intimate partner violence and household food insecurity.

Table A5. Mean differences in Z-scores by maternal characteristics: results from random effects linear regression models with repeat measures

	Weight-for-age (WAZ)			Length-for-age (LAZ)			Head circumference-for-age (HCAZ)		
	Crude mean difference (95% CI)	Adjusted mean difference (95% CI)[a]	*p*-value for interaction with HIV[b]	Crude mean difference (95% CI)	Adjusted mean difference (95% CI)[a]	*p*-value for interaction[b]	Crude mean difference (95% CI)	Adjusted mean difference (95% CI)[a]	*p*-value for interaction[b]
Maternal HIV	−0.34 (−0.47; −0.21)	−0.28 (−0.41; −0.15)	–	−0.20 (−0.34; −0.06)	−0.13 (−0.28; 0.11)	–	−0.30 (−0.43; −0.16)	−0.26 (−0.40; −0.11)	–
Hazardous drinking at first antenatal visit[c]	−0.41 (−0.59; −0.23)	−0.30 (−0.49; −0.10)	0.83	−0.40 (−0.60; −0.21)	−0.32 (−0.52; −0.11)	0.36[d]	−0.26 (−0.46; −0.06)	−0.16 (−0.36; 0.05)	0.90
Hazardous drinking at 12 months' visit[c]	−0.16 (−0.34; 0.03)	–	0.54	−0.15 (−0.37; 0.08)	–	0.87	−0.06 (−0.30; 0.17)	–	0.40
Categories of HIV and hazardous drinking (HD)[c,e]									
No HIV, no HD	Ref	–	–	Ref			Ref		
HIV, no HD	−0.28 (−0.41; −0.14)	–	–	−0.11 (−0.26; 0.04)	–	–	−0.27 (−0.41; −0.12)	–	–
No HIV, HD	−0.28 (−0.65; 0.08)	–	–	−0.20 (−0.57; 0.16)	–	–	−0.20 (−0.62; 0.22)	–	–
HIV and HD	−0.61 (−0.81; −0.40)	–	–	−0.52 (−0.74; −0.29)	–	–	−0.44 (−0.67; −0.21)	–	–
Intimate partner violence at first antenatal visit[c]	−0.23 (−0.41; −0.05)	−0.09 (−0.27; 0.10)	0.21	−0.21 (−0.42; −0.01)	−0.08 (−0.29; 0.13)	0.45	−0.18 (−0.38; 0.02)	−0.07 (−0.28; 0.14)	0.19
Intimate partner violence at 12 months' visit[c]	−0.19 (−0.41; −0.02)	–	0.70	−0.14 (−0.41; 0.14)	–	0.90	−0.05 (−0.32; 0.23)	–	0.25
Categories of HIV and intimate partner violence (IPV)[c,e]									
No HIV, no IPV	Ref	–	–	Ref	–		Ref	–	–
HIV, no IPV	−0.35 (−0.49; −0.21)	–	–	−0.19 (−0.35; −0.04)	–	–	−0.31 (−0.46; −0.16)	–	–
No HIV, IPV	−0.34 (−0.68; 0.000)	–	–	−0.29 (−0.61; 0.04)	–	–	−0.33 (−0.70; 0.04)	–	–
HIV and IPV	−0.43 (−0.63; −0.23)	–	–	−0.32 (−0.57; −0.07)	–	–	−0.35 (−0.58; −0.11)	–	–

(*Continued*)

Table A5. (Continued)

	Weight-for-age (WAZ)			Length-for-age (LAZ)			Head circumference-for-age (HCAZ)		
	Crude mean difference (95% CI)	Adjusted mean difference (95% CI)[a]	*p*-value for interaction with HIV[b]	Crude mean difference (95% CI)	Adjusted mean difference (95% CI)[a]	*p*-value for interaction[b]	Crude mean difference (95% CI)	Adjusted mean difference (95% CI)[a]	*p*-value for interaction[b]
Probable depression at first antenatal visit[c]	−0.01 (−0.24; 0.22)	–	0.60	0.01 (−0.23; 0.26)	–	0.43	0.04 (−0.21; 0.30)	–	0.60
Probable depression at 12 months' visit[c]	0.02 (−0.28; 0.33)	–	0.41	0.09 (−0.23; 0.42)	–	0.16	0.13 (−0.18; 0.45)	–	0.38
Household food insecurity[c]	−0.11 (−0.24; 0.02)	−0.06 (−0.19; 0.06)	0.068[f]	−0.04 (−0.19; 0.11)	−0.01 (−0.16; 0.14)	0.89	−0.11 (−0.27; 0.05)	−0.07 (−0.28; 0.14)	0.14[g]
Categories of HIV and household food insecurity (HFIS)[c,e]									
No HIV, no HFIS	Ref	–	–	Ref	–	–	Ref	–	–
HIV, no HFIS	−0.26 (−0.42; −0.10)	–	–	−0.19 (−0.38; −0.02)	–	–	−0.21 (−0.39; −0.04)	–	–
No HIV, has HFIS	0.06 (−0.14; 0.25)	–	–	0.00 (−0.20; 0.20)	–	–	0.05 (−0.18; 0.29)	–	–
HIV, has HFIS	−0.43 (−0.61; −0.25)	–	–	−0.22 (−0.42; −0.02)	–	–	−0.39 (−0.59; −0.18)	–	–

Abbreviations: CI, confidence interval; HCAZ, head circumference for age Z-scores; HD, hazardous drinking; HFIS, household food insecurity; IPV, intimate partner violence; LAZ, length-for-age Z-scores; WAZ, weight-for-age Z-scores.

[a]Regression models built following multiple imputation, using Rubin's rules; random effects linear regression with adjustment for clustering; multivariable model includes maternal HIV status, hazardous drinking at first antenatal visit, IPV at first antenatal visit and household food insecurity at 12 months.

[b]Using bivariable models including HIV and variable of interest, with interaction terms for each of HIV-IPV, HIV-hazardous drinking and HIV-food insecurity, respectively.

[c]Definitions: hazardous drinking, AUDIT-C score ≥3; intimate partner violence any physical, sexual or psychological violence (WHO VAW questionnaire) as reported at study enrolment; probable depression, EPDS score ≥13; household food insecurity—at risk of or has household food insecurity per questionnaire at 12 months postpartum (questionnaire adapted from the Household Food Insecurity Access Scale [HFIAS], Food and Nutrition Technical Assistance Project [FANTA] and the Community Childhood Hunger Indentification Project Index [CCHIP]).

[d]β3, −0.20 (95% CI −0.64; 0.23).

[e]Combination categorical variables use data from the first antenatal visit except for household food insecurity measures.

[f]β3 (measure of additive interaction effect), −0.23 (95% CI −0.48; 0.017).

[g]β3, −0.22 (95% CI −0.52; 0.07).

Table A6. Relative odds (odds ratios) for underweight, stunting and microcephaly over time, by maternal characteristics: results from random effects logistic regression models with repeat measures

	Underweight (WAZ< −2)			Stunted (LAZ<−2)			Microcephaly (HCAZ<−2)		
Crude OR (95% CI)	aOR (95% CI)[a]	*p*-value for interaction[b]	Crude OR (95% CI)	aOR (95% CI)[a]	*p*-value for interaction[b]	Crude OR (95% CI)	aOR (95% CI)[a]	*p*-value for interaction[b]	
Maternal HIV	2.73 (1.37; 5.44)	2.20 (1.09; 4.46)	–	2.27 (0.14; 3.66)	1.97 (1.19; 3.27)	–	2.31 (1.29; 4.15)	2.06 (1.13; 3.75)	–
Hazardous drinking at first antenatal visit[c]	3.44 (1.57; 8.00)	2.53 (1.098; 5.93)		2.28 (1.24; 4.17)	1.74 (0.93; 3.26)	0.97	1.69 (0.85; 3.37)	1.20 (0.63; 2.28)	0.47
Hazardous drinking at 12 months' visit[c]	1.90 (0.76; 4.76)	–		1.41 (0.66; 2.98)	–	0.72	1.20 (0.48; 3.02)	–	0.45
Categories of HIV and hazardous drinking (HD)[c,d]									
No HIV, no HD	Ref	–	–	Ref	–	–	Ref	–	–
HIV, no HD	2.31 (1.11; 4.82)	–	–	2.00 (1.20; 3.33)	–	–	2.00 (1.06; 3.79)	–	–
No HIV, HD	3.57 (0.84; 15.13)	–	–	1.78 (0.39; 8.02)	–	–	0.66 (0.09; 5.07)	–	–
HIV and HD	6.01 (2,22; 16.22)	–	–	3.67(1.82; 7.38)	–	–	2.95 (1.32; 6.58)	–	–
Intimate partner violence at first antenatal visit[c]	1.65 (0.70; 3.91)	1.04 (0.40; 2.65)		1.64 (0.87; 3.10)	1.12 (0.58; 2.16)	0.52	1.68 (0.80; 3.53)	1.29 (0.64; 2.64)	0.98
Intimate partner violence at 12 months' visit[c]	2.55 (0.94; 6.90)	–		1.74 (0.80; 3.76)	–	0.24	2.21 (0.89; 5.47)	–	0.22
Categories of HIV and intimate partner violence (IPV)[c,d]									
No HIV, no IPV	Ref	–	–	Ref	–	–	Ref	–	–
HIV, no IPV	3.25 (1.54; 6.88)	–	–	2.32 (1.38; 3.88)	–	–	2.19 (1.17; 4.11)	–	–
No HIV, IPV	4.17 (0.86; 20.2)	–	–	1.99 (0.49; 8.01)	–	–	1.35 (0.30; 6.05)	–	–
HIV and IPV	2.93 (1.02; 8.42)	–	–	2.75 (1.31; 5.77)	–	–	3.02 (1.23; 7.43)	–	–

(Continued)

Table A6. (Continued)

	Underweight (WAZ< −2)			Stunted (LAZ<−2)			Microcephaly (HCAZ<−2)		
Crude OR (95% CI)	aOR (95% CI)[a]	*p*-value for interaction[b]	Crude OR (95% CI)	aOR (95% CI)[a]	*p*-value for interaction[b]	Crude OR (95% CI)	aOR (95% CI)[a]	*p*-value for interaction[b]	
Probable depression at first	1.54			0.80		0.29	1.61	–	0.40
antenatal visit[c]	(0.50; 4.75)			(0.32; 1.95)			(0.59; 4.39)		
Probable depression at 12	2.11	–		0.93	–	0.97	2.17	–	0.34
months' visit[c]	(0.56; 7.94)			(0.29; 2.97)			(0.71; 6.62)		
Household food insecurity[c]	1.87	1.50	0.02	1.28	1.11	0.69	2.11	1.85	0.37
	(0.91; 3.82)	(0.76; 2.95)		(0.77; 2.13)	(0.67; 1.83)		(1.16; 3.81)	(1.04; 3.27)	
Categories of HIV and household food insecurity (HFIS)[c,d]									
No HIV, no HFIS	Ref	–	–	Ref	–	–	Ref	–	–
HIV, no HFIS	1.35	–	–	2.06	–	–	1.74	–	–
	(0.56; 3.27)			(1.12; 3.80)			(0.81; 3.75)		
No HIV, has HFIS	0.41	–	–	0.99	–	–	1.27	–	–
	(0.11; 1.52)			(0.42; 2.32)			(0.45; 3.57)		
HIV, has HFIS	3.90	–	–	2.56	–	–	3.92	–	–
	(1.67; 9.11)			(1.34; 4.86)			(1.79; 8.59)		

Abbreviations: aOR, adjusted odds ratio; CI, confidence interval; HCAZ, head circumference for age Z-scores; HD, hazardous drinking; HFIS, household food insecurity; IPV, intimate partner violence; LAZ, length-for-age Z-scores; OR, odds ratio; WAZ, weight-for-age Z-scores.

[a]Regression models built following multiple imputation, using Rubin's rules; random effects logistic regression with adjustment for clustering; multivariable model includes maternal HIV status, HD at first antenatal visit, IPV at first antenatal visit and household food insecurity at 12 months.

[b]Using multivariable model as above, with interaction terms for HIV-IPV, HIV-HD and HIV-food insecurity.

[c]Definitions: HD, AUDIT-C score ≥3; intimate partner violence any physical, sexual or psychological violence (WHO VAW questionnaire) as reported at study enrolment; probable depression, EPDS score ≥13; household food insecurity—at risk of or has household food insecurity per questionnaire at 12 months postpartum (questionnaire adapted from the Household Food Insecurity Access Scale [HFIAS], Food and Nutrition Technical Assistance Project [FANTA] and the Community Childhood Hunger Indentification Project Index [CCHIP]).

[d]Combination categorical variables use data from the first antenatal visit except for household food insecurity measures.

Table A7. Relative odds (odds ratios) for infectious morbidity events, by maternal characteristics: results from logistic regression models

	Ever hospitalised		Ever hospitalised, beyond first 72 hours of life		Any infectious-cause hospitalisation between ages 7 days and 3 months		Any ambulatory respiratory tract illness reported during follow-up		Any ambulatory diarrhoeal illness reported during follow-up	
n/N (%)	378/872 (44%)		104/872 (12%)		40/849 (15%)		135/872 (15%)		307/872 (35%)	
	OR (95% CI)	aOR (95% CI)	OR (95% CI)	aOR (95% CI)	OR (95% CI)	aOR (95% CI)	OR (95% CI)	aOR (95% CI)	OR (95% CI)	aOR (95% CI)
Maternal HIV	1.02 (0.78; 1.34)	1.02 (0.77; 1.36)	2.42 (1.55; 3.80)	2.24 (1.41; 3.58)	3.30 (1.55; 7.02)	3.31 (1.52; 7.21)	2.85 (1.89; 4.28)	2.51 (1.64; 3.85)	1.12 (0.85; 1.48)	0.89 (0.66; 0.21)
Hazardous drinking at first antenatal visit[a]	0.76[b] (0.53; 1.01)	0.69 (0.47; 1.02)	1.37 0.82; 2.28)	0.93 (0.53; 1.61)	0.85 (0.35; 2.07)	0.47 (0.18; 1.28)	1.50 (0.96; 2.36)	1.09 (0.67; 1.78)	1.58 (1.10; 2.27)	1.42 (0.96; 2.09)
Hazardous drinking at 12 months' visit[a]	0.64 (0.41; 1.01)	–	0.74 (0.36; 1.52)	–	0.47 (0.11; 1.94)	–	0.96 (0.53; 1.72)	–	1.14 (0.73; 1.79)	–
Categories of HIV and hazardous drinking (HD)[a,c]										
No HIV, no HD	Ref	–	Ref	–	Ref	–	Ref	–	Ref	–
HIV, no HD	1.01 (0.75; 1.35)	–	2.35 (1.45; 3.81)	–	3.36 (0.54; 7.31)	–	2.80 (1.80; 4.35)	–	0.91 (0.67; 1.24)	–
No HIV, HD	0.46 (0.20; 1.05)	–	0.90 (0.20; 3.98)	–	n/a	–	1.17 (0.34; 4.07)	–	0.69 (0.30; 1.60)	–
HIV and HD	0.86 (0.57; 1.32)	–	2.58 (1.39; 4.78)	–	2.32 (0.78; 6.40)	–	3.14 (1.80; 5.50)	–	1.81 (1.19; 2.75)	–
Intimate partner violence at first antenatal visit[a]	1.03[d] (0.71; 1.49)	1.12 (0.75; 1.65)	1.46 (0.87; 2.46)	1.25 (0.72; 2.15)	1.43 (0.64; 3.19)	1.33 (0.58; 3.06)	1.43 (0.89; 2.30)	1.13 (0.68; 1.87)	1.85 (0.69; 1.89)	1.78 (1.20; 2.64)
Intimate partner violence at 12 months' visit[a]	1.19 (0.73; 1.95)	–	0.87 (0.39; 1.91)	–	1.24 (0.42; 360)	–	0.99 (0.52; 1.90)	–	1.15 (0.69; 1.89)	–
Categories of HIV and intimate partner violence (IPV)[a,c]										
No HIV, no IPV	Ref	–	Ref	–	Ref	–	Ref	–	Ref	–
HIV, no IPV	0.98 (0.73; 1.30)	–	2.73 (1.67; 4.47)	–	3.38 (1.50; 7.63)	–	2.53 (1.64; 3.90)	–	0.95 (0.70; 1.29)	–
No HIV, IPV	0.78 (0.37; 1.64)	–	2.62 (0.93; 7.39)	–	1.47 (0.18; 12.15)	–	0.32 (0.04; 2.39)	–	1.19 (0.56; 2.51)	–
HIV and IPV	1.10 (0.71; 1.71)	–	2.63 (1.34; 5.14)	–	3.58 (1.26; 10.13)	–	3.27 (1.85; 5.78)	–	2.05 (1.31; 3.19)	–

(*Continued*)

Table A7. (Continued)

n/N (%)	Ever hospitalised 378/872 (44%) OR (95% CI)	aOR (95% CI)	Ever hospitalised, beyond first 72 hours of life 104/872 (12%) OR (95% CI)	aOR (95% CI)	Any infectious-cause hospitalisation between ages 7 days and 3 months 40/849 (15%) OR (95% CI)	aOR (95% CI)	Any ambulatory respiratory tract illness reported during follow-up 135/872 (15%) OR (95% CI)	aOR (95% CI)	Any ambulatory diarrhoeal illness reported during follow-up 307/872 (35%) OR (95% CI)	aOR (95% CI)
Probable depression at first antenatal visit[a]	1.02[e] (0.63; 1.64)	–	0.62 (0.26; 1.46)	–	1.22 (0.43; 3.54)	–	0.63 (0.29; 1.34)	–	1.03 (0.63; 1.69)	–
Probable depression at 12 months' visit[a]	2.39 (1.25; 4.56)	–	0.67 (0.23; 1.93)	–	0.94 (0.22; 4.05)	–	2.54 (1.17; 2.55)	–	1.52 (0.82; 2.81)	–
Household food insecurity[a]	1.02[f] (0.76; 1.39)	1.03 (0.76; 1.41)	1.39 (0.90; 2.15)	1.24 (0.79; 2.15)	1.53 (0.78; 3.00)	1.34 (0.68; 2.67)	1.73 (1.17; 2.55)	1.52 (1.02; 2.27)	1.45 (1.07; 1.97)	1.42 (1.03; 1.94)
Categories of HIV and household food insecurity (HFIS)[a,c]										
No HIV, no HFIS	Ref	–	Ref	–	Ref	–	Ref	–	Ref	–
HIV, no HFIS	1.11 (0.78; 1.58)	–	1.70 (0.96; 2.99)	–	2.69 (1.00; 7.22)	–	3.46 (1.95; 6.13)	–	1.13 (0.78; 1.63)	–
No HIV, has HFIS	1.27 (0.80; 2.01)	–	0.65 (0.25; 1.66)	–	0.95 (0.18; 5.07)	–	2.43 (1.18; 4.99)	–	1.62 (1.03; 2.59)	–
HIV, has HFIS	0.97 (0.66; 1.43)	–	2.68 (1.53; 4.70)	–	3.87 (1.45; 10.29)	–	4.38 (2.43; 7.87)	–	1.48 (1.00; 2.19)	–

Note: Regression models built following multiple imputation, using Rubin's rules; random effects logistic regression with adjustment for clustering; multivariable model includes maternal HIV status, HD at first antenatal visit, IPV at first antenatal visit and household food insecurity at 12 months.

Abbreviations: aOR, adjusted odds ratio; CI, confidence interval; HD, hazardous drinking; HFIS, household food insecurity; IPV, intimate partner violence; n/a, not applicable—inestimable due to zero cells; OR, odds ratio.

[a]Definitions: HD, AUDIT-C score ≥3; intimate partner violence any physical, sexual or psychological violence (WHO VAW questionnaire) as reported at study enrolment; probable depression, EPDS score ≥13; household food insecurity—at risk of or has household food insecurity per questionnaire at 12 months postpartum (questionnaire adapted from the Household Food Insecurity Access Scale [HFIAS], Food and Nutrition Technical Assistance Project [FANTA] and the Community Childhood Hunger Indentification Project Index [CCHIP]).

[b]*p*-Value for interactions of HIV with *HD* at enrolment: ever hospitalised, $p = 0.19$; ever hospitalised beyond the first 72 hours of life, $p = 0.81$; any infectious cause hospitalisation between ages of 7 days and 3 months, *p* = *n/a*; any ambulatory respiratory tract illness during follow-up, $p = 0.95$; any ambulatory diarrhoeal illness during follow-up, $p = 0.03$.

[c]Combination categorical variables use data from the first antenatal visit except for household food insecurity measures.

[d]*p*-Value for interactions of HIV with *intimate partner violence* at enrolment: ever hospitalised, $p = 0.40$; ever hospitalised beyond the first 72 hours of life, $p = 0.10$; any infectious cause hospitalisation between ages of 7 days and 3 months, $p = 0.78$; any ambulatory respiratory tract illness during follow-up, $p = 0.19$; any ambulatory diarrhoeal illness during follow-up, $p = 0.18$.

[e]*p*-Value for interactions of HIV with *maternal probable depression* at enrolment: ever hospitalised, $p = 0.42$; ever hospitalised beyond the first 72 hours of life, inestimable due to zero cell; any infectious cause hospitalisation between ages of 7 days and 3 months, n/a; any ambulatory respiratory tract illness during follow-up, $p = 0.61$; any ambulatory diarrhoeal illness during follow-up, $p = 0.98$.

[f]*p*-Value for interactions of HIV with *food insecurity* at 12 months: ever hospitalised, $p = 0.23$; ever hospitalised beyond the first 72 hours of life, $p = 0.10$; any infectious cause hospitalisation between ages of 7 days and 3 months, $p = 0.66$; any ambulatory respiratory tract illness during follow-up, $p = 0.14$; any ambulatory diarrhoeal illness during follow-up, $p = 0.49$.

Table A8. Mean differences in BSID-III composite developmental scores, by maternal characteristics, at approximately 12 months of age: results from linear regression models

	Composite cognitive score			Composite motor score		
	Crude mean difference (95% CI)	Adjusted mean difference (95% CI)[a]	*p*-value for interaction[b]	Crude mean difference (95% CI)	Adjusted mean difference (95% CI)[a]	*p*-value for interaction[b]
Maternal HIV	0.50 (−1.88; 2.88)	0.74 (−1.80; 3.28)	–	0.67 −1.74; 3.08)	1.78 (−0.77; 4.31)	–
Hazardous drinking at first antenatal visit[c]	0.07 (−3.09; 3.23)	−0.01 (−3.40; 3.37)	0.81	−2.16 (−5.38; 1.05)	−1.99 (−5.40; 1.43)	0.50
Hazardous drinking at 12 months' visit[c]	−0.36 (−3.91; 3.20)	–	0.20	−2.14 (−5.70; 1.42)	–	0.14
Categories of HIV and hazardous drinking (HD)[c,d]						
No HIV, no HD	Ref	–	–	Ref	–	–
HIV, no HD	0.40 (−2.30; 3.10)	–	–	1.56 (−1.15; 4.27)	–	–
No HIV, HD	−0.69 (−6.36; 4.98)	–	–	−1.02 (−6.69; 4.65)	–	–
HIV and HD	0.56 (−3.21; 4.33)	–	–	−1.88 (−5.73; 1.96)	–	–
Intimate partner violence at first antenatal visit[c]	−0.06 (−3.62; 3.49)	−0.12 (−3.83; 3.60)	0.42	−2.17 (−5.74; 1.39)	−1.79 (−5.50; 1.91)	0.41
Intimate partner violence at 12 months' visit[c]	1.86 (−2.68; 6.39)	–	0.49	−0.98 (−5.53; 3.57)	–	0.49
Categories of HIV and intimate partner violence (IPV)[c,d]						
No HIV, no IPV	Ref	–	–	Ref	–	–
HIV, no IPV	0.15 (−2.45; 2.74)	–	–	1.32 (−1.30; 3.94)	–	–
No HIV, IPV	−2.07 (−7.85; 3.70)	–	–	−0.76 (−6.55; 5.02)	–	–
HIV and IPV	1.15 (−3.30; 5.60)	–	–	−2.20 (−6.66; 2.25)	–	–
Probable depression at first antenatal visit[c]	2.24 (−2.19; 6.68)	–	0.16	3.23 (−1.21; 7.67)	–	0.16
Probable depression at 12 months' visit[c]	3.13 (−2.80; 9.05)	–	0.43	−2.69 (−8.50; 3.11)	–	0.43
Household food insecurity[c]	−1.12 (−3.54; 1.30)		0.01	−2.62 (−5.06; −0.18)	−2.67 (−5.16; −0.18)	0.01
Categories of HIV and household food insecurity (HFIS)[c,d]						
No HIV, no HFIS	Ref	–	–	Ref	–	–
HIV, no HFIS	3.25 (0.14; 6.36)	–	–	3.47 (0.34; 6.60)	–	–
No HIV, has HFIS	1.61 (−1.71; 4.93)	–	–	−0.24 (−3.57; 3.06)	–	–
HIV, has HFIS	−1.50 (−4.70; 1.70)	–	–	−2.58 (−5.82; 0.65)	–	–

Abbreviations: BSID-III, Bayley Scales of Infant Development, 3rd edition; CI, confidence interval; HD, hazardous drinking; HFIS, household food insecurity; IPV, intimate partner violence.

[a]Regression models built following multiple imputation, using Rubin's rules; random effects linear regression with adjustment for clustering; multivariable model includes maternal HIV status, hazardous drinking at 12 months' visit, IPV at 12 months' visit and household food insecurity at 12 months' visit.

[b]Using multivariable model as above, with interaction terms for HIV-IPV, HIV-HD and HIV-food HFIS.

[c]Definitions: HD, AUDIT-C score ≥3; intimate partner violence any physical, sexual or psychological violence (WHO VAW questionnaire) as reported at study enrolment; probable depression, EPDS score ≥13; household food insecurity—at risk of or has household food insecurity per questionnaire at 12 months postpartum (questionnaire adapted from the Household Food Insecurity Access Scale [HFIAS], Food and Nutrition Technical Assistance Project [FANTA] and the Community Childhood Hunger Indentification Project Index [CCHIP]).

[d]Combination categorical variables use data from the first antenatal visit except for household food insecurity measures.

Table A9. Relative odds (odds ratios) of developmental delay (BSID-III composite score <85), by maternal characteristics, at approximately 12 months of age: results from logistic regression models

	Cognitive delay Overall prevalence: 34/515 (7%)			Motor delay Overall prevalence: 32/510 (6%)		
	Crude OR (95% CI)	Aor (95% CI)[a]	*p*-value for interaction[b]	Crude OR (95% CI)	aOR (95% CI)[a]	*p*-value for interaction[b]
Maternal HIV	2.19 (1.08; 4.44)	2.23 (1.06; 4.68)		2.01 (0.97; 4.12)	1.39 (0.64; 3.05)	–
Hazardous drinking at first antenatal visit[c]	0.66 (0.23; 1.93)	0.46 (0.15; 1.43)	n/a	2.18 (0.97; 4.90)	1.35 (0.55; 3.32)	0.25
Hazardous drinking at 12 months' visit[c]	1.56 (0.62; 3.93)	–	0.29	1.67 (0.66; 4.23)	–	0.32
Categories of HIV and hazardous drinking (HD)[c,d]						
No HIV, no HD	Ref	–	–	Ref	–	–
HIV, no HD	2.29 (1.08; 4.83)	–	–	2.18 (0.94; 5.06)	–	–
No HIV, HD	n/a	–	–	3.51 (0.91; 13.5)	–	–
HIV and HD	1.35 (0.43; 4.24)	–	–	2.85 (1.01; 8.05)	–	–
Intimate partner violence at first antenatal visit[c]	0.94 (0.32; 2.77)	0.88 (0.29; 2.72)	0.82	3.03 (1.33; 6.89)	2.35 (1.09; 4.90)	0.75
Intimate partner violence at 12 months' visit[c]	0.37 (0.05; 2.81)	–	n/a	1.35 (0.39; 4.66)	–	0.77
Categories of HIV and intimate partner violence (IPV)[c,d]						
No HIV, no IPV	Ref	–		Ref	–	–
HIV, no IPV	2.31 (1.10; 4.88)	–		1.61 (0.70; 3.75)	–	–
No HIV, IPV	0.94 (0.12; 7.53)	–		2.14 (0.45; 10.21)	–	–
HIV and IPV	1.65 (0.45; 6.06)	–		4.68 (1.72; 12.72)	–	–
Probable depression at first antenatal visit[c]	1.21 (0.35; 4.16)	–	n/a	0.80 (0.18; 3.50)	–	n/a
Household food insecurity[c]	2.27 (1.13; 4.59)	2.09 (1.02; 4.29)	0.34	2.67 (1.29; 5.54)	2.31 (1.09; 4.90)	0.50
Categories of HIV and household food insecurity (HFIS)[c,d]						
No HIV, no HFIS	Ref	–		Ref	–	–
HIV, no HFIS	1.33 (0.46; 3.84)	–		1.26 (0.40; 3.96)	–	–
No HIV, has HFIS	1.34 (0.44; 4.12)	–		1.84 (0.62; 5.47)	–	–
HIV, has HFIS	3.69 (1.54; 8.84)	–		3.96 (1.58; 9.91)	–	–

Abbreviations: BSID-III, Bayley Scales of Infant Development, 3rd edition; CI, confidence interval; HD, hazardous drinking; HFIS, household food insecurity; IPV, intimate partner violence; n/a, not applicable due to zero cell.

[a]Regression models built following multiple imputation, using Rubin's rules; random effects linear regression with adjustment for clustering; multivariable model includes maternal HIV status, HD at 12 months' visit, IPV at 12 months' visit and household food insecurity at 12 months' visit.

[b]Using multivariable model as above, with interaction terms for HIV-IPV, HIV-HD and HIV-HFIS.

[c]Definitions: hazardous drinking, AUDIT-C score ≥3; intimate partner violence any physical, sexual or psychological violence (WHO VAW questionnaire) as reported at study enrolment; probable depression, EPDS score ≥13; household food insecurity—at risk of or has household food insecurity per questionnaire at 12 months postpartum (questionnaire adapted from the Household Food Insecurity Access Scale [HFIAS], Food and Nutrition Technical Assistance Project [FANTA] and the Community Childhood Hunger Indentification Project Index [CCHIP]).

[d]Combination categorical variables use data from the first antenatal visit except for household food insecurity measures.

Table A10. Interaction on the additive and/or the multiplicative scale: testing variation in exposure effects by strata of maternal HIV status, HD, intimate partner violence and household food insecurity, using crude risk ratios and epidemiological measures of interaction

		Risk of outcome per subgroup (proportions)[a]				Measure of interaction	Relative risk (RR) of each exposure group versus non-exposed group			Additional measures of interaction[b]	
		Double exposure	Exposure to HIV only	Exposure to second factor only	No exposure	Interaction contrast[c]	Double exposure (RR11)	HIV exposure only (RR10)	Other factor exposure only (RR01)	$RERI_{RR}$	Multiplicative interaction measure
Out-come	Second exposure variable	p_{11}	p_{10}	p_{01}	p_{00}	$p_{11} - p_{10} - p_{01} + p_{00}$	p_{11}/p_{00}	p_{10}/p_{00}	p_{01}/p_{00}	$RR_{11} - RR_{10} - RR_{01} + 1$	$(RR_{11})/(RR_{10}RR_{01})$
Ever underweight[d]	Hazardous drinking[e]	0.16	0.10	0.10	0.06	0.02	2.57	1.59	1.62	0.36	1.00
	IPV[f]	0.12	0.11	0.12	0.06	−0.06	1.95	2.05	1.89	−0.98	0.50
	HFIS[g]	0.17	0.09	0.04	0.08	**0.12**	2.19	0.49	1.2	**1.49**	**3.68**
Ever stunted[d]	Hazardous drinking[e]	0.26	0.18	0.17	0.13	0.03	2.03	1.32	1.44	0.27	1.07
	IPV[f]	0.23	0.19	0.16	0.13	0.01	1.79	1.23	1.50	0.06	0.97
	HFIS[g]	0.20	0.21	0.15	0.13	−0.03	1.47	1.10	1.56	−0.19	0.86
Ever microcephalic[d]	Hazardous drinking[e]	0.05	0.05	0.03	0.03	0.00	1.64	1.06	1.52	0.06	1.02
	IPV[f]	0.04	0.05	0.06	0.03	−0.04	1.38	2.14	1.76	−1.52	0.37
	HFIS[g]	0.07	0.04	0.05	0.02	0.01	3.50	2.40	1.85	0.25	0.79
Ever admitted	Hazardous drinking[e]	0.41	0.45	0.27	0.44	**0.14**	0.92	0.60	1.01	0.31	**1.52**
	IPV[f]	0.46	0.43	0.37	0.43	**0.09**	1.07	0.86	0.99	0.21	**1.25**
	HFIS[g]	0.43	0.46	0.49	0.44	−0.08	0.97	1.12	1.03	0.19	0.84
Ever admitted beyond first 72 hours	Hazardous drinking[e]	0.17	0.16	0.07	0.07	0.02	2.34	0.92	2.18	0.25	**1.17**
	IPV[f]	0.16	0.16	0.16	0.07	−0.09	2.39	2.36	2.45	−1.42	0.41
	HFIS[g]	0.21	0.15	0.06	0.09	**0.09**	2.35	0.64	1.67	**1.03**	**2.19**
Infectious cause hospitalisation <3 months of age	Hazardous drinking[e]	0.052	0.08	Null	0.02	0.00	2.17	n/a	3.21	−0.04	n/a
	IPV[f]	0.07	0.07	0.03	0.02	−0.01	3.50	1.48	3.33	−0.29	0.72
	HFIS[g]	0.09	0.07	0.02	0.02	0.02	4.40	0.95	3.35	**1.10**	**1.38**
Ever ambulatory respiratory infection[h]	Hazardous drinking[e]	0.23	0.21	0.10	0.09	0.01	2.66	1.15	2.42	0.08	0.95
	IPV[f]	0.28	0.21	0.03	0.09	**0.10**	2.70	0.34	2.23	**1.13**	**3.56**
	HFIS[g]	0.26	0.22	0.15	0.07	−0.04	3.801	2.20	3.19	−0.59	0.54

(Continued)

Table A10. (Continued)

Out-come	Second exposure variable	Risk of outcome per subgroup (proportions)[a]				Measure of interaction	Relative risk (RR) of each exposure group versus non-exposed group			Additional measures of interaction[b]	
		Double exposure	Exposure to HIV only	Exposure to second factor only	No exposure	Interaction contrast[c]	Double exposure (RR11)	HIV exposure only (RR10)	Other factor exposure only (RR01)	$RERI_{RR}$	Multiplicative interaction measure
		p_{11}	p_{10}	p_{01}	p_{00}	$p_{11} - p_{10} - p_{01} + p_{00}$	p_{11}/p_{00}	p_{10}/p_{00}	p_{01}/p_{00}	$RR_{11} - RR_{10} - RR_{01} + 1$	$(RR_{11})/(RR_{10}RR_{01})$
Ever ambulatory diarrhoeal illness[h]	Hazardous drinking[e]	0.49	0.32	0.27	0.34	**0.24**	1.42	0.78	0.94	0.70	**1.95**
	IPV[f]	0.50	0.32	0.37	0.33	**0.14**	1.51	1.12	0.96	0.43	**1.41**
	HFIS[g]	0.44	0.37	0.47	0.35	−0.06	1.25	1.344	1.07	−0.16	0.87
Cognitive delay[i]	Hazardous drinking[e]	0.07	0.11	Null	0.05	0.01	1.34	n/a	2.16	.18	n/a
	IPV[f]	0.08	0.10	0.04	0.05	−0.02	1.63	0.96	2.22	−0.54	0.77
	HFIS[g]	0.01	0.06	0.06	0.04	−0.06	0.33	1.33	1.33	−1.33	0.19
Motor delay[i]	Hazardous drinking[e]	0.10	0.08	0.12	0.04	−0.06	2.69	3.20	2.10	−1.61	0.40
	IPV[f]	0.17	0.07	0.09	0.04	0.03	4.07	2.02	1.58	**1.46**	**1.27**
	HFIS[g]	0.13	0.05	0.07	0.04	0.06	3.62	1.81	1.27	**1.54**	**1.57**

Note: The most prominent estimates suggesting positive interaction on additive or relative scale are given in **bold**.
Abbreviations: HD, hazardous drinking; HFIS, household food insecurity; IPV, intimate partner violence; $RERI_{RR}$, relative excess risk due to interaction, also referred to as interaction contrast ratio (ICR); RR, relative risk.
[a]Number of outcomes per category, over number of children in each category; denominators vary slightly due to missing data for some variables (missing data HD, $n = 2$; IPV, $n = 4$; food insecurity, $n = 134$).
[b]Additive interaction: $RERI_{RR}$ <0, negative interaction; $RERI_{RR}$>0, positive interaction ($RERI_{RR}$ measures departure from additive effects, in terms of direction but not magnitude as baseline group risks may vary between groups); multiplicative interaction: measure <1, negative interaction; >1, positive interaction (measures the extent to which, on the RR scale, the effect of both exposures together exceeds the product of the effects of the two exposures considered separately); both measures rounded to the nearest decimal.
[c]Commonly used measure for interaction on the additive scale: if >0, positive interaction; <0, negative interaction; rounded to nearest decimal.
[d]Binary growth indicators, summarised over all follow-up; weight-for-age, length-for-age and head circumference-for-age Z-scores <−2, respectively.
[e]HD, defined as Alcohol use disorders identification test (AUDIT-C) score ≥3 versus <3, first antenatal visit.
[f]Any physical, sexual or psychological violence reported at first antenatal visit.
[g]Has or is at risk of food insecurity, based on questionnaire adapted from the Household Food Insecurity Access Scale (HFIAS), Food and Nutrition Technical Assistance Project (FANTA) and the Community Childhood Hunger Indentification Project Index (CCHIP).
[h]Maternal self-report using administered questionnaire derived from DHS surveys at each study visit: presumed lower respiratory tract infection defined as cough plus fever plus difficulty in breathing; diarrhoeal illness defined as loose or increased frequency of stools.
[i]Composite score <85 per domain, using Bailey Scales of Infant Development, 3rd edition.
